# Comparative gut proteomics study revealing adaptive physiology of Eurasian spruce bark beetle, *Ips typographus* (Coleoptera: Scolytinae)

**DOI:** 10.3389/fpls.2023.1157455

**Published:** 2023-11-21

**Authors:** Muhammad Zubair Ashraf, Kanakachari Mogilicherla, Gothandapani Sellamuthu, Valentina Siino, Fredrik Levander, Amit Roy

**Affiliations:** ^1^Faculty of Forestry and Wood Sciences, Czech University of Life Sciences Prague, Prague, Czechia; ^2^Department of Immunotechnology, Lund University, Lund, Sweden; ^3^National Bioinformatics Infrastructure Sweden (NBIS), Science for Life Laboratory, Lund University, Lund, Sweden

**Keywords:** conifer pests, Scolytinae, *Ips typographus* (L.), comparative proteomics, DAPs, digestion, detoxification, enzyme assay

## Abstract

The bark beetle, *Ips typographus* (L.), is a major pest of Norway spruce, *Picea abies* (L.), causing enormous economic losses globally. The adult stage of the *I. typographus* has a complex life cycle (callow and sclerotized); the callow beetles feed ferociously, whereas sclerotized male beetles are more aggressive and pioneers in establishing new colonies. We conducted a comparative proteomics study to understand male and female digestion and detoxification processes in callow and sclerotized beetles. Proteome profiling was performed using high-throughput liquid chromatography-mass spectrometry. A total of >3000 proteins were identified from the bark beetle gut, and among them, 539 were differentially abundant (fold change ±2, FDR <0.05) between callow and sclerotized beetles. The differentially abundant proteins (DAPs) mainly engage with binding, catalytic activity, anatomical activity, hydrolase activity, metabolic process, and carbohydrate metabolism, and hence may be crucial for growth, digestion, detoxification, and signalling. We validated selected DAPs with RT-qPCR. Gut enzymes such as NADPH-cytochrome P450 reductase (CYC), glutathione S-transferase (GST), and esterase (EST) play a crucial role in the *I. typographus* for detoxification and digesting of host allelochemicals. We conducted enzyme activity assays with them and observed a positive correlation of CYC and GST activities with the proteomic results, whereas EST activity was not fully correlated. Furthermore, our investigation revealed that callow beetles had an upregulation of proteins associated with juvenile hormone (JH) biosynthesis and chitin metabolism, whereas sclerotized beetles exhibited an upregulation of proteins linked to fatty acid metabolism and the TCA cycle. These distinctive patterns of protein regulation in metabolic and functional processes are specific to each developmental stage, underscoring the adaptive responses of *I. typographicus* in overcoming conifer defences and facilitating their survival. Taken together, it is the first gut proteomic study comparing males and females of callow and sclerotized *I. typographus*, shedding light on the adaptive ecology at the molecular level. Furthermore, the information about bark beetle handling of nutritionally limiting and defence-rich spruce phloem diet can be utilized to formulate RNAi-mediated beetle management.

## Introduction

Tree-killing bark beetles are a significant disturbance in biomes and temperate climates, impacting the function, composition, and structure of forests (Vindstad et al., 2019; Vindstad et al., 2020; Hlásny et al., 2021b). In *Europe*, *Ips typographus* (L.) is a significant insect pest of Norway spruce *Picea abies* (L.) H. Karst trees, causing substantial economic losses (Christiansen and Bakke, 1988; Hlásny et al., 2021a). The Norway spruce is critical species for the wood and timber industry because the damage caused by *I. typographus* outbreaks can have a cascade effect on forest economies and the global timber market, as well as environmental impacts, carbon sink, and well-being of humans (Seidl et al., 2014; Grégoire et al., 2015; Aranda et al., 2017; Morris et al., 2017; Montagné-Huck and Brunette, 2018; Hlásny et al., 2019; Schuldt et al., 2020; Senf et al., 2020). The bark beetle epidemic was reported in various *European* nations, including *Germany*, *France*, and *Austria*, but the *Czech Republic* became the epicentre of the bark beetle attack (Hlásny et al., 2019; Gao et al., 2020; Jandl, 2020; Toth et al., 2020; Hlásny et al., 2021a; Senf and Seidl, 2021; Sommerfeld et al., 2021). In 2015, a severe outbreak started in Czech Republic, and the losses have exceeded over the last two centuries. This outbreak can be called ecological collapse or failure of spruce-oriented management. Adult host colonization and overwintering may be critical in increasing the number of bark beetle attacks and their economic impact (Senf and Seidl, 2021). Superior knowledge of the molecular mechanisms underlying its adaptation to host allelochemicals can facilitate its management via RNAi (Joga et al., 2021; Mogilicherla and Roy, 2023).

Researchers have applied multiple approaches to explore insect resistance and host adaptation mechanisms, such as measuring biochemical and genetic differences, altering protein expression, etc. Biochemical techniques, including colourimetry, successfully identify and quantify detoxification enzymes such as *esterase*, *glutathione S-transferase*, *cytochrome*, etc. (Bosch-Serra et al., 2021). Transcriptomic approaches reflect organism biology because they allow the investigation of genes that are expressed in a tissue or organism under certain conditions. However, levels of proteins and enzymes are sometimes hard to predict from corresponding mRNA expression, whereas proteomics can deliver more reliable information about the physiological state of an organism (Huber, 2003). In the mountain pine beetle (*Dendroctonus ponderosae Hopkins*), the integrated analysis of expressed sequence tag (EST) and proteomics data showed several proteins were to be involved in the reproduction and stress physiology of insect survival during the early stages of the host colonization phase of their life cycle (Pitt et al., 2014). The proteomics and transcriptomics approaches were used to study the overwintering physiology (insects living under the tree’s bark must endure protracted periods of extreme cold) and early host colonization (insects must surpass host defences to procreate) of the mountain pine beetle (*D. ponderosae Hopkins*) (Huber and Robert, 2016). Quantitative metabolome, proteome, and transcriptome analysis of juvenile hormone III treated male/female midgut and fat body tissues of mountain pine beetle (*D. ponderosae Hopkins*) revealed JH III stimulates frontalin biosynthesis in males and trans-verbenol biosynthesis in females (Keeling et al., 2016). Recently, our group performed a thorough analysis of the *I. typographus* transcriptome data to comprehend physiology and adaptation and unravel the dynamics of gene expression in various life stages [larvae (L1, L2, and L3), pupa, callow, and sclerotized adult] and male/female tissues (gut, fat body, and head) from callow and sclerotized beetles (Naseer et al., 2023). Unfortunately, no proteomic study on the Eurasian tree-killing bark beetle delineates the digestive and adaptive physiology during the adult stage. We are interested in the protein expression dynamics in the guts of *I. typographus* males and females of callow (freshly emerged from the pupa and appeared yellow) and sclerotized (fully matured and appeared black) beetles to follow up existing transcriptomics studies and understand beetle adaptive physiology better. Callow beetles perform maturation feeding and need to digests tons of plant materials whereas sclerotized beetles (males for *I. typographus*) are pioneers in finding a new host for colonization and are later accompained by females to produce the next generation of beetles. Hence, these two stages are the focal point of the current proteomic study. The present study identified differentially abundant proteins (DAPs) in the guts of male and female adult beetles at callow and sclerotized stages. It will aid in understanding detoxification, digestion, signalling, and growth mechanisms in this economically significant forest pest of spruce and serve as a foundation for future studies.

## Materials and methods

### Insect collection, rearing, and sample collection

Naturally infested spruce stems with *I. typographus* were collected from the forest (Kostelec nad Černými lesy, 50°00’07.2”N 14°50’56.3”E, Czech Republic, under School forest enterprise, semi-warm and dry area) and kept in the university insect breeding room at the Czech university of life sciences Prague, Czech Republic. After emerging, beetles were reared on spruce logs in plastic boxes under laboratory conditions of 25 ± 2°C and 70% relative humidity. Logs were peeled, and callow beetles were collected from spruce logs after 5 weeks of infestation, whereas sclerotized beetles were collected after emerging from spruce logs, and both stages were sexed into males and females based on pronotum hair density (Schlyter and Cederholm, 1981). A total of 500 males and females of callow and sclerotized beetles were collected and stored at -80°C for further study.

### Dissection and beetle gut collection

The frozen beetles were thawed and dissected. The guts were collected in an ice-cold 1× phosphate buffer solution (PBS) with a protease inhibitor cocktail. Fifteen beetle guts were pooled for each replicate, and four replicates were prepared for each sample.

### Protein extraction, quantification, and SDS PAGE

Gut samples were homogenized in 50 µL of lysis buffer (100 mM PBS, 5mM DTT, and protease inhibitor cocktail) and centrifuged at 13000 rpm at room temperature for 15 minutes. The supernatant was separated, the protein concentration was determined using the Bradford assay (Bradford, 1976), and the standard was a serial dilution of bovine serum albumin (R^2 ^= 0.942). Each biological replicate contains 50 µg of total gut protein mixed with 2× loading buffer and boiled for 5 minutes before loading onto a 5% SDS PAGE stacking gel as per preoptimized protocol (Roy et al., 2014; Roy and Das, 2015). The SDS-PAGE gel was run for 10 mins until the total protein band reached the resolving gel (10%). Subsequently, protein gels were stained using Coomassie brilliant blue R250. The protein bands from the gel were cut into 1mm pieces and stored in 0.5x destaining solution (ethanol, glacial acetic acid, and proteomic-graded water) at 4°C after separation for 10 mins at a constant 50V (**Supplementary Figure 1**).

### In-gel digestion

Each gel lane containing a single unseparated total protein (50 µg) band was cut and washed twice with acetonitrile (ACN) and ammonium bicarbonate (AMBIC) (**Supplementary Figure 1**). Gel pieces were then reduced and alkylated by 10 mM DL-dithiothreitol (DTT) and 55 mM iodoacetamide (IAA) correspondingly. Proteins were digested by porcine-modified trypsin in a 1:50 w/w ratio with incubation overnight at 37°C. Finally, peptides were extracted from gel pieces with 70% ACN/5% formic acid (FA) and dried out in the SpeedVac (Thermo Fisher Scientific) (Shevchenko et al., 2006; Roy et al., 2014).

### Mass spectrometry analysis

The dried peptides were dissolved in 0.1% FA and quantified using nanodrop (Thermo Fisher Scientific). Approximately 400 ng peptides per sample were separated by liquid chromatography on an Evosep One system (Evosep, Odense), connected to a QExactive HF-X mass spectrometer (Thermo Fisher Scientific). A 15 cm long fused silica capillary (75 μm* 16 cm Pico Tip Emitter, New Objective) was utilized as the analytical column, packed in-house with C18 material ReproSil-Pur 1.9 μm (Dr Maisch, GmbH, Germany). Peptides were separated using the Whisper 58 min gradient pre-programmed for 20 samples per day. The mass spectrometer was operated in positive ion mode using data-dependent acquisition with an automated gain control (AGC) target value of 3×10^6^ ions and a maximum fill time of 50 ms in a scan range of 375 to 1500 m/z for the entire MS scans. A top-20 method was used for fragmentation (collision energy: 40V) by higher energy collision-induced dissociation (HCD) using an isolation window of 1.2 m/z and a target value of 1×10^5^ ions with a maximum injection period of 20 ms. MS/MS spectra were collected at a resolution of 15,000 full-width half-maximum (FWHM).

### Mass spectrometry data processing

The resulting RAW files were processed for label-free quantification using MaxQuant, version 1.6.17.0 (www.maxquant.org). The bark beetle fasta file created in-house from the published genome of *I. typographus* (Powell et al., 2021) was used as the search database. Cysteine carbamidomethylation was set as a fixed modification, whereas methionine oxidation was selected as a variable modification. Default settings were used, including protein level filtering at FDR < 0.01. The search results and raw data have been deposited at ProteomeXchange via the PRIDE partner repository with protein accession PXD035407 (Project Name: Bark beetle gut proteomics study). The search parameters are available with the deposited data.

### Proteomics data analysis

Protein group quantity values from MaxQuant were normalized using Cyclic Loess-normalization in NormalyzerDE version 1.10.0 (Willforss et al., 2018). The log_2_-transformed normalized data were utilized for visualization and statistical tests to identify differentially abundant peptides (Bengtsson et al., 2014). The differential abundance analysis was performed using LIMMA Empirical Bayes statistics in NormalyzerDE. We filtered DAPs with an Adjusted p-value <0.05 and a fold difference ±2 times and sorted them for further analysis and verification (**Supplementary Excel File 1**). Functional analysis of DAPs was carried out using gene ontology (GO) analysis, and the DAPs were grouped according to the biological process, molecular function, and cellular component. Further, functional annotations of DAPs were classified through KEGG ontology assignments using the Blast2GO platform to study the biological functions and pathways using preoptimized pipeline (Conesa et al., 2005; Roy et al., 2016).

### Protein preparation and measurement of enzyme activity

Adult beetles (sclerotized and callow) were collected from logs and sexed into male and female. Four individual replicates were prepared, and for each replicate, 10 beetles were dissected, extracted in the guts, and homogenized into 100 µL sodium phosphate buffer (50 mM, pH 7) depending on the requirement of the enzymatic concentration (Smagghe and Tirry, 2001). Initial homogenization was performed manually with the help of a homogenizer at 4°C in the Eppendorf tube. Supernatants were separated by centrifuging homogenized extracts for 15 mins at 12000 g and 4°C (Z32 HK, Hermle, Newyork, USA) (Berrada et al., 1994). The supernatant was collected individually and stored at -20°C before further analysis. Protein quantification was done using the Bradford method (Bradford, 1976). The colorimetric method and substrates for each enzymatic assay were used to analyze the activity of enzymes related to detoxification and digestion (GST, CYC, and EST) in beetles. All the enzymatic activities were studied based on changes in absorbance at 30°C using a VICTOR3™ Multilabel microplate reader (PerkinElmer Life and Analytical Sciences, Madrid, Spain) (Lehninger, 1950). To evaluate the impact of protein concentration on enzyme activity, we conducted an enzymatic experiment using different concentrations of enzyme sources (0.5, 1, 2, and 4 g of total protein) at varying time intervals (1 to 10 minutes) following the addition of the substrate.

To measure glutathione S-transferase (GST) activity, 20 µl of enzyme source and 170 µl of 5mM GSH (dissolved in 50 mM sodium phosphate buffer) were added to a 96-well transparent plate. The plate was preincubated at 30°C for 60 minutes, followed by the addition of 10 µl of 30mM CDNB (dissolved in 100% ethanol); the colour development was monitored at 340 nm every minute for a 30-minute incubation, maintaining a temperature of 25-30°C (Habig and Jakoby, 1981; Bosch-Serra et al., 2021). The enzyme activity was quantified as a nmol substrate conjugated.min^-1^.mg^-1^ protein, utilizing a CDNB molar extinction coefficient of 9.6 mM^-1^ × cm^-1^ (Habig and Jakoby, 1981).

To assess Cytochrome P450 Reductase (CYC) activity, 20 µL of enzyme extract was combined with 160 µL of NADPH solution (containing 0.6 mM NADP, 2.8 mM G6P, and 0.28 units of G6PD) in a 96-well plate, followed by a preincubation at 30°C for 30 minutes. Subsequently, 20 µL of 1 mM cytochrome C solution (dissolved in 50 mM sodium phosphate buffer, pH 7) was added, and the resulting colour change was monitored at 550 nm at one-minute intervals for a duration of up to 25 minutes (Ortego et al., 1999; Bosch-Serra et al., 2021). The activity of CYC was quantified as a nmol substrate conjugated.min ^-1^.mg^-1^ protein, employing a cytochrome C molar extinction coefficient of 27.6 mM^-1^ × cm^-1^ (Margoliash and Frohwirt, 1959). The enzymatic activity was measured in cytochrome P450 equivalent unit min–1 mg–1 of the total extracted protein.

The esterase (EST) activity was performed at 25°C in a total volume of 200 μl, comprising 2 μl of 100 mM substrate (p-nitrophenyl acetate), 10 μl of enzyme source, 8 μl of ethanol (99%), and 180 μl of Tris–HCl buffer (50 mM, pH 8.0). The reaction mixture without the addition of any enzyme served as the control. The experiment was carried out at 37°C for 5 minutes, and the assessment was performed using a colourimetric assay by measuring the absorbance at 410 nm (Li et al., 2020). Enzymatic activity was defined as one unit when 1 µmol of p-nitrophenol was released min ^-1^. mg^-1^ protein, determined by referring to the α-naphthol standard curve (R^2 ^= 0.952).

### Linking gene expression at transcriptomics and protein level

Differentially expressed transcripts (DET) from our previously published data (Naseer et al., 2023) and DAPs (from the current study) above fold change ±2 and adjusted *P*-value ≤0.05 cutoff values were used to determine the correlation between proteomic and transcriptomic level expression of (**Supplementary Excel File 2**) on *I. typographus* gut genes. The Venn diagram was designed to find the genes commonly expressed between transcriptome and proteome analyses. A pie chart was also made with commonly expressed genes to identify the percentage of genes associated with detoxification, digestion, defence, signalling, and growth.

### RNA extraction and cDNA synthesis

Four replicates from each male and female of callow and sclerotized beetles were prepared following preoptimized protocol (Sellamuthu et al., 2022), and each replicate contains five beetle guts. Total RNA was isolated from the males and females of the callow and sclerotized beetle’s guts using TRIzol^®^ (Invitrogen, Carlsbad, CA) according to the manufacturer’s instructions. Further, to remove the DNA, extracted RNA was treated with TURBO™ DNase (Ambion, USA). The cDNA was synthesized from 1 μg RNA using High-Capacity cDNA Reverse Transcription kits (Applied Biosystems-Life Technologies) following the manufacturer’s instructions and stored at -20°C. Before the RT-qPCR reaction, the cDNA samples were diluted ten times with nuclease-free water.

### Validation of DAPs by RT- qPCR

RT-qPCR reaction was performed in StepOnePlus™ Real-Time PCR System (Applied Biosystems, USA) with four independent biological replicates from guts of callow and sclerotized beetles. The master mix contains 5 µl SYBR^®^ Green PCR Master Mix (Applied Biosystems, USA), 1 µl of diluted cDNA, 1 µl of gene-specific primers, and makes the final volume of 10 µl with nuclease-free water (Invitrogen, USA). All the samples were amplified under the following conditions: pre-degeneration at 95°C for 10 min, followed by 40 cycles of denaturation for 15 s at 95°C, and 60°C for 1 min. The primer’s specificity was confirmed by melt curve analysis using default parameters by a gradual increase in temperature from 60°C to 95°C. The 40S ribosomal protein S3-A was employed for data normalization as an internal control gene (Sellamuthu et al., 2022). The 2^-ΔΔCt^ method was used for qRT-PCR data validation (Livak and Schmittgen, 2001).

## Results

### Proteomic analysis

**Figure 1:**
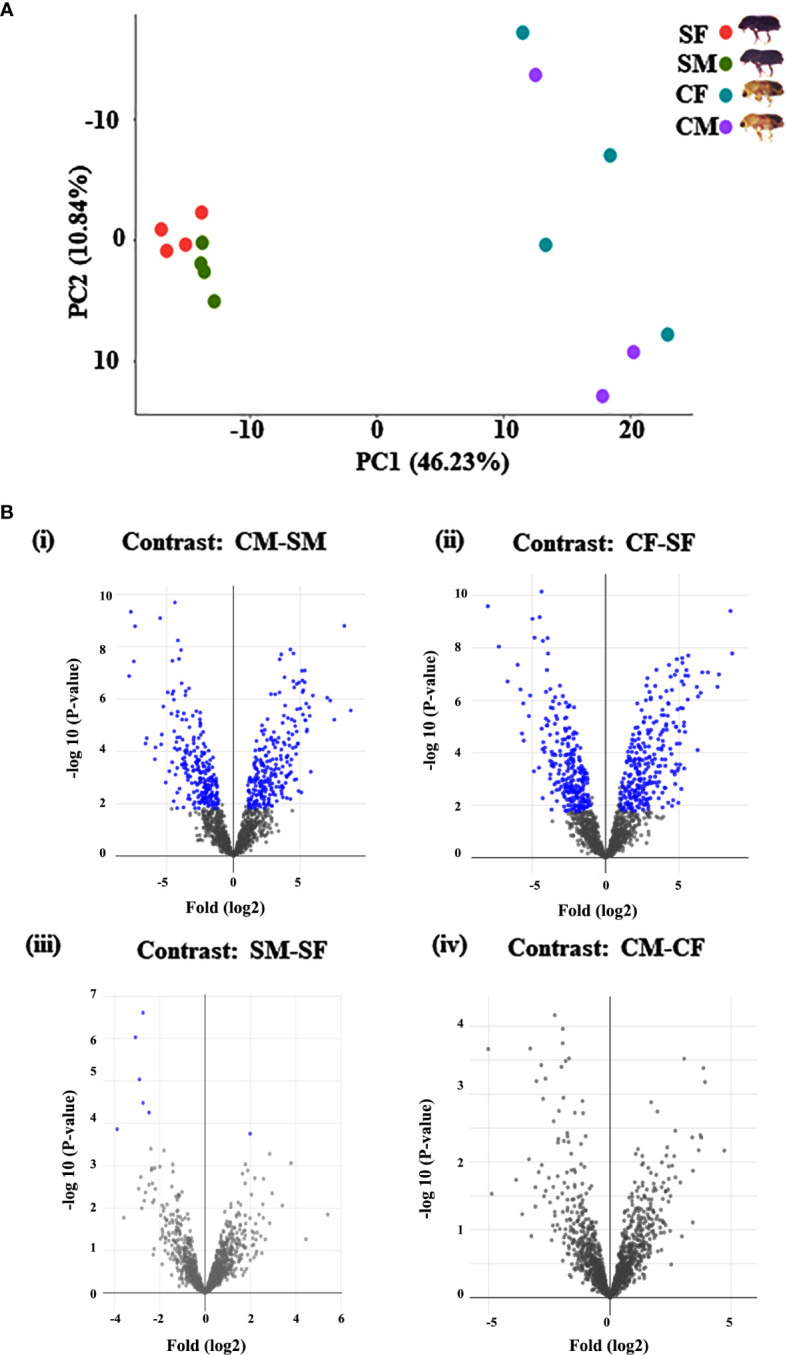
Graphical representation of quantitative proteomics data. **(A)** The PCA plot represents the uniformity of each group of samples of callow and sclerotized stages. Percentages indicate the total variance explained by the first and second PC. Dots of the same colour show the four biological replicates of each sample (*callow males have only three replicates). **(B)** Proteins are ranked in a volcano plot according to their statistical P-value (y-axis) and their relative abundance ratio (log2 fold change) between four comparisons (x-axis). Off-centred spots are those that vary the most between the groups. Blue dots represent the differentially abundant proteins based on Log2FC 1 (fold change 2) and FDR corrected p value <0.05 cut-offs. CM, callow male; CF, callow female; SM, sclerotized male; SF, sclerotized female.

**Figure 2:**
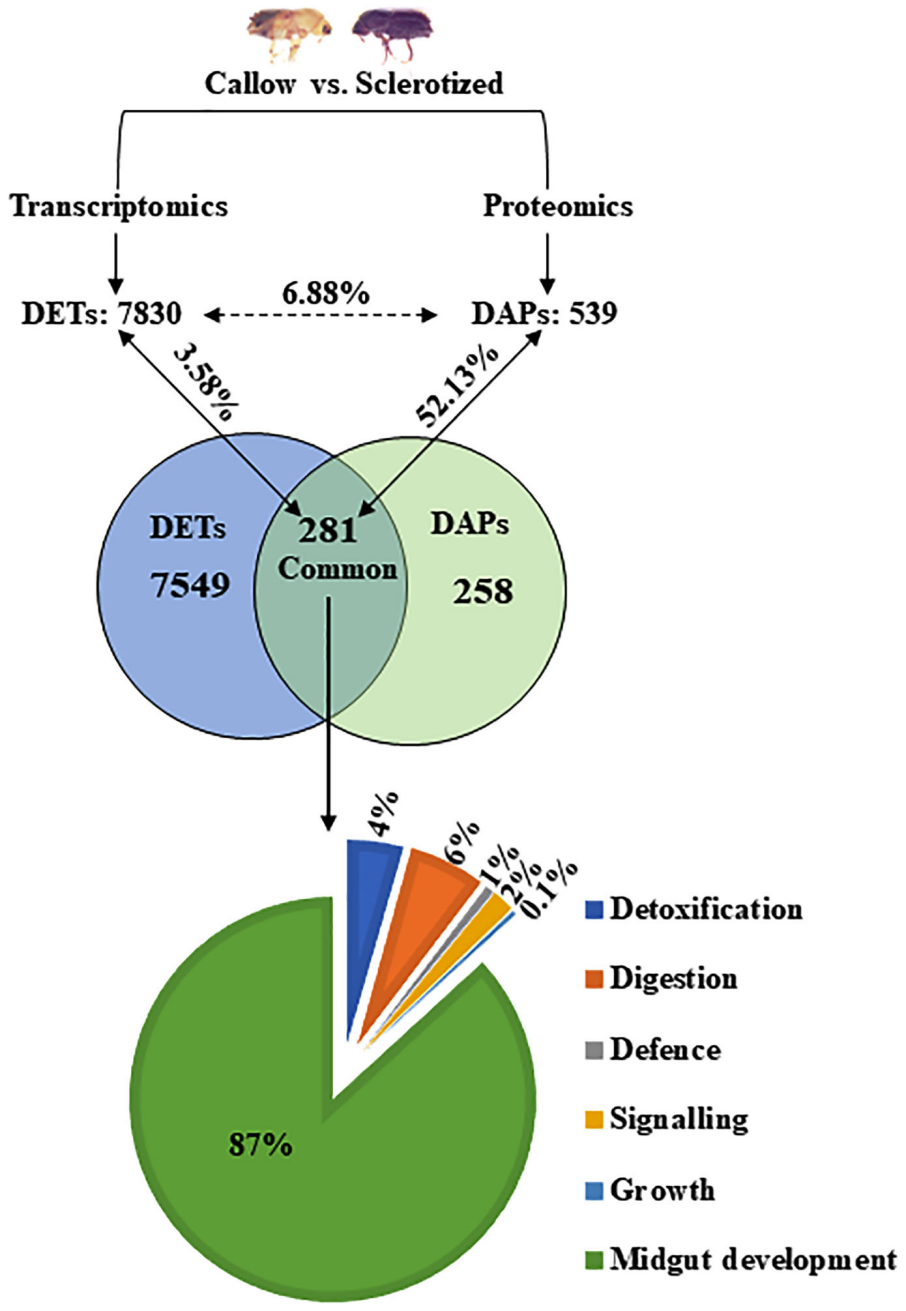
Correlation between DAPs and DETs in the gut (callow vs. sclerotized). The numerical value in each circle represents the number of transcripts or proteins, including identified transcripts and proteins related to gut expression and transcripts/proteins related to gut expression, respectively. DAPs, differentially abundant proteins; DETs, differentially expressed transcripts.

**Figure 3:**
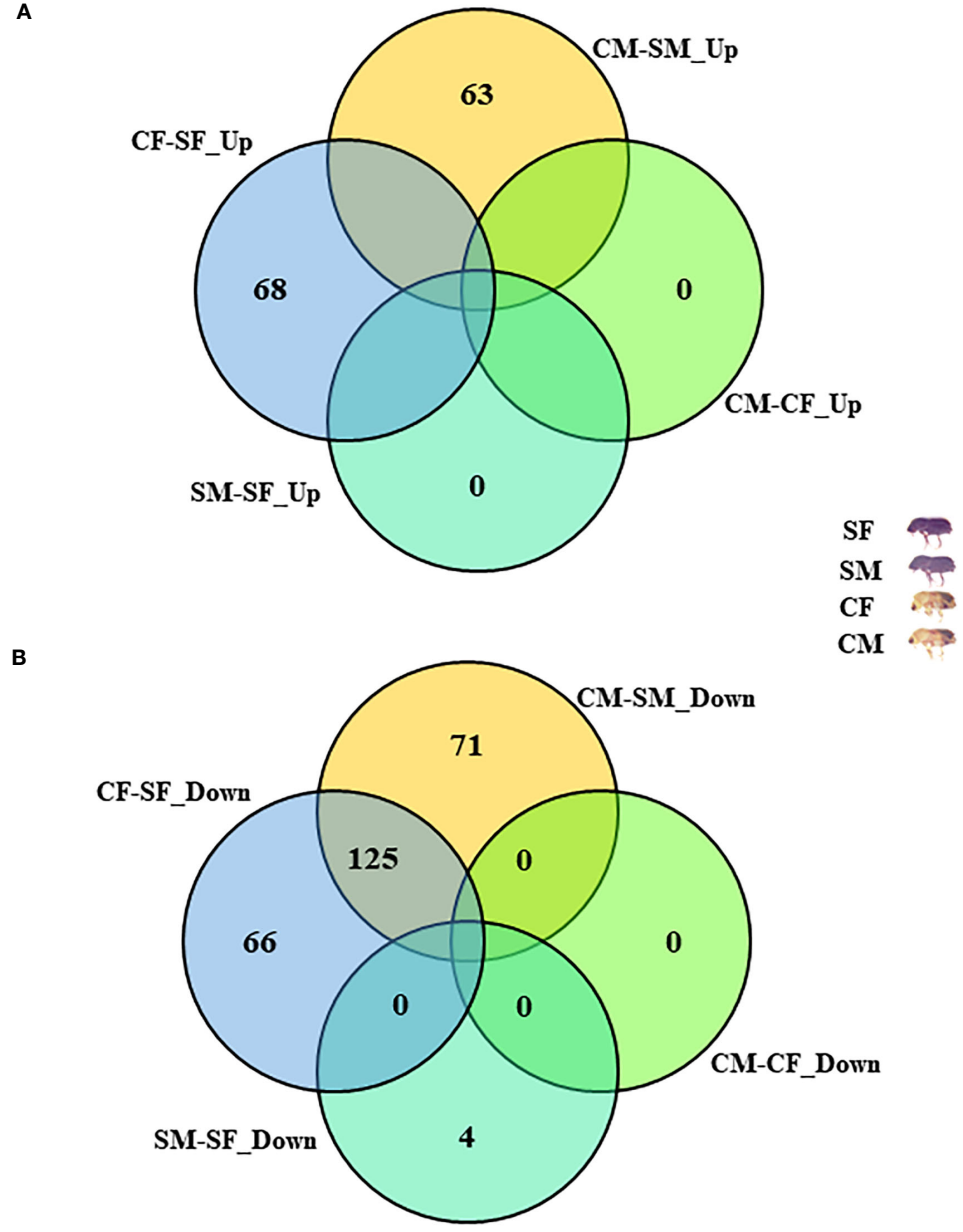
Comparative analysis with differentially abundant proteins (DAPs). **(A)** Distribution of common upregulated DAPs between four comparisons. **(B)** Distribution of common downregulated DAPs between four comparisons. CM, callow male; CF, callow female; SM, sclerotized male; SF, sclerotized female; DAPs, differentially abundant proteins.

The *I. typographus* males and females of callow and sclerotized beetle gut samples were used for the proteomic study (**Supplementary Figure 1**). Total proteins were prepared, digested, and analyzed through LC-MS analysis, identifying 3061 proteins. 539 DAPs were identified after deploying the cutoff (fold change ± 2 and FDR p-value < 0.05) (**Figures 1A, B**). Furthermore, 281 commonly regulated genes were discovered by comparing the differentially abundant proteins with pre-existing gut transcriptome data (**Figure 2**), signifying their physiological importance in the bark beetle gut. Most proteins were found to be differentially regulated between callow and sclerotized stages rather than between males and females of the same stages (**Figures 3A, B**), which is understandable. Furthermore, based on Gene Ontology (GO) classification, the DAPs were classified into three functional categories: biological process, molecular function, and cellular components (**Supplementary Figure 2**).

### Functional clustering for DAPs

**Figure 4:**
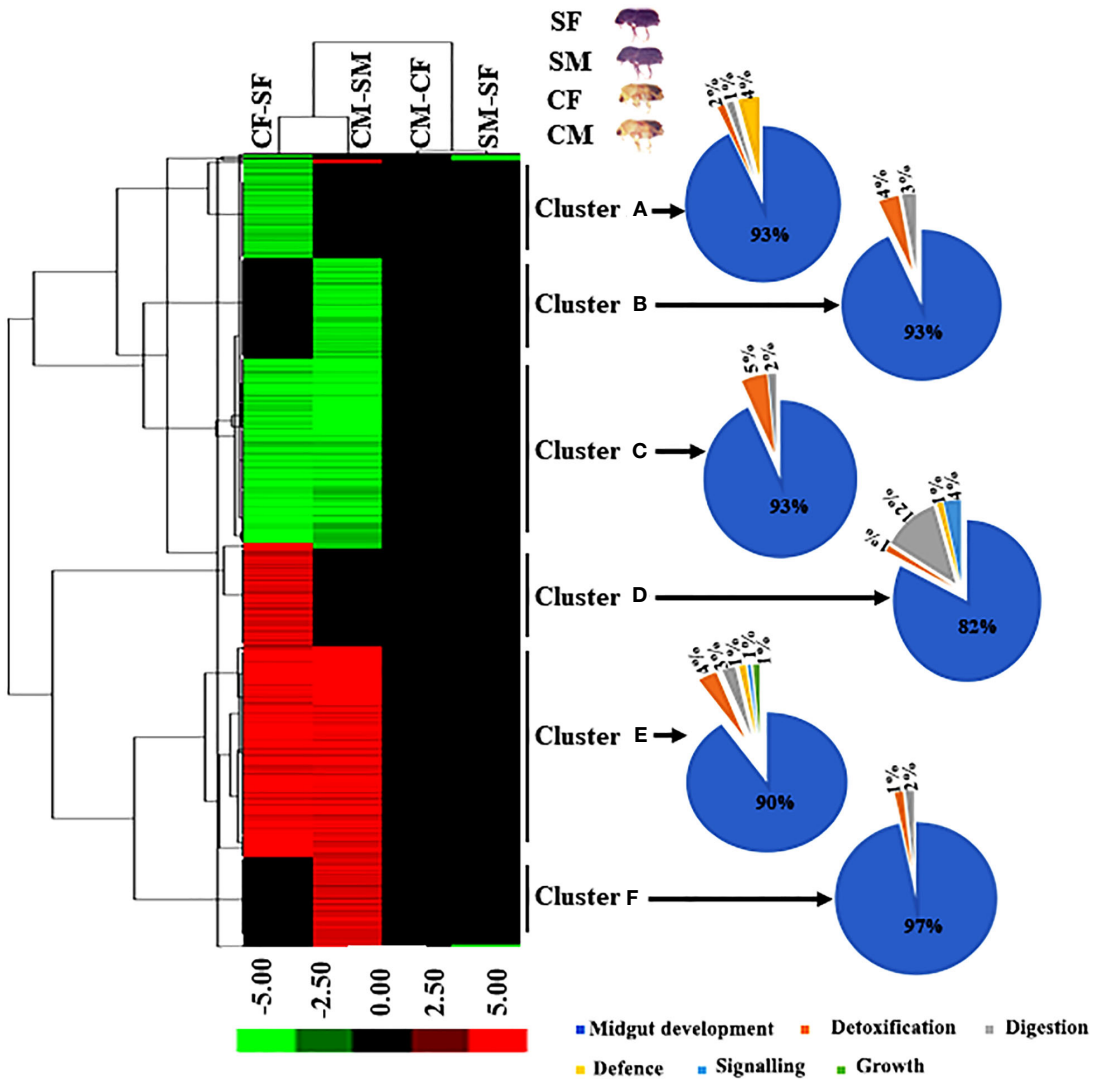
Hierarchical cluster analysis of DAPs in four comparisons. The letters (A-F) indicate major groups identified by cluster analysis. Red colour indicates upregulation (>2.0 fold), green colour indicates downregulation (<-2.0 fold), and black indicates no change as compared to respective controls. The Venn diagram represents the cluster-wise percentage of proteins related to detoxification, digestion, defence, signalling, and growth. CM, callow male; CF, callow female; SM, sclerotized male; SF, sclerotized female; DAPs, differentially abundant proteins.

We performed hierarchical clustering to identify the expression of DAPs between males and females of callow and sclerotized beetles. Hierarchical clustering was carried out using the average linkage approach with Euclidean distance based on log fold change data using Cluster 3.0 to depict the expression pattern of differentially abundant peptides. (Eisen et al., 1998). Significant up and down-regulation was observed in all DAPs between callow and sclerotized females (CF vs SF) and callow and sclerotized males (CM vs SM) comparisons (**Figure 4**). Although the DAPs almost overlapped, fold enrichment significantly differed between callow and sclerotized beetles, indicating that the magnitude of protein expression is sex-specific in response to ecological role and environmental challenges.

### DAPs vs. DETs comparison

539 DAPs from the proteomic study and 7830 DETs (differentially expressed transcripts) from the transcriptomic study (Naseer et al., 2023) were compared to relate the gene expression in callow vs. sclerotized beetle gut. The DAPs (539) and DETs (7830) were chosen to build a Venn diagram leading to the identification of 281 commonly expressed transcripts/proteins (**Figure 2**). The percentage of commonly expressed DETs was 3.58% (281 out of 7830), and DAPs were 52.13% (281 out of 539). Out of the identified 281 common ones, genes associated with detoxification (4%), digesting (6%), defence (1%), signalling (2%), and growth (0.1) were also represented on a pie chart (**Figure 2**).

### DAPs validation by RT-qPCR and enzymatic assay

**Figure 5:**
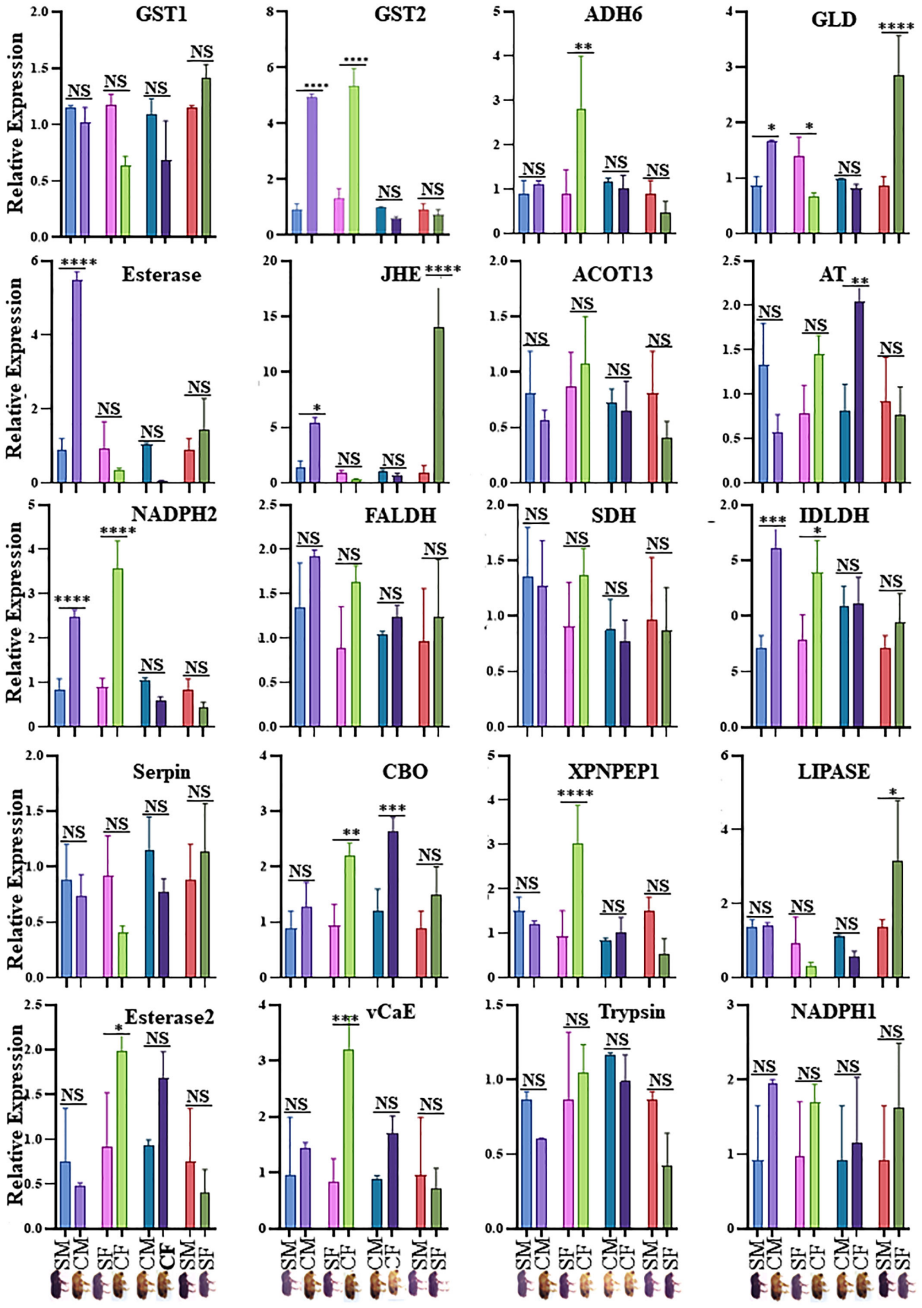
Validation of expression of genes obtained from proteomics analysis in four comparisons. SM- sclerotized male; CM-callow male; SF- sclerotized female; CF-callow female. The X-axis represents the sample names, and the Y-axis represents the relative expression. * P < 0.01; ** P < 0.001; *** P < 0.0006; **** P < 0.0001, NS- non-significant. GST1, Glutathione S-transferase, GST2, Glutathione S-transferase-like, ADH6, Alcohol dehydrogenase [NADP (+)]-like, GLD, Glucose dehydrogenase [acceptor], Esterase: Esterase B1-like, JHE, Juvenile hormone esterase, ACOT13, Acyl-coenzyme A thioesterase 13-like, AT, Antitrypsin-like isoform X4, NADPH2, NADH-ubiquinone oxidoreductase 75 kDa subunit, mitochondrial, FALDH, Fatty aldehyde dehydrogenase-like, SDH, Hypothetical protein D910_04066, IDLDH, IDLDH_IPSPIRecName, Full= Ipsdienol dehydrogenase, Serpin, Serine protease inhibitor 27A-like, CBQ, Carboxypeptidase Q-like, XPNPEP1, Xaa-Pro aminopeptidase 1, LIPASE, Lipase 3-like, Esterase2, Esterase E4-like, vCaE, Venom carboxylesterase-6-like, Trypsin, Trypsin precursor, NADPH1, NADPH, adrenodoxin oxidoreductase, mitochondrial. CM, callow male; CF, callow female; SM, sclerotized male; SF, sclerotized female.

**Figure 6:**
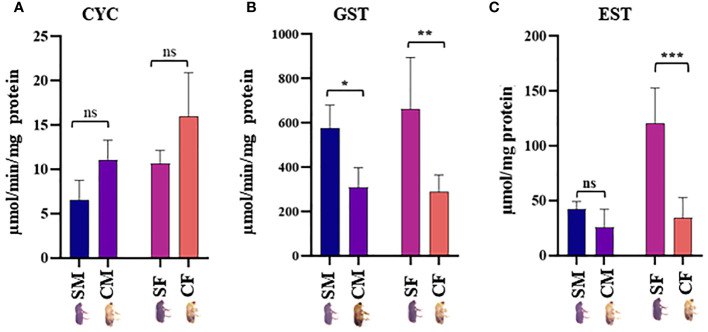
Enzymatic assay. **(A)** Expression levels of NADPH-cytochrome P450 reductase (CYC), **(B)** glutathione-S-transferase (GST), and **(C)** Esterases (EST) in males and females gut tissues of callow and sclerotized beetles (Mean± SE; P < 0.05). CM, callow male; CF, callow female; SM, sclerotized male; SF, sclerotized female. * denotes a significant difference between values from each other at * p < 0.05, ** p < 0.01, *** p < 0.001, and "ns" denotes non-significant.

Twenty physiologically important DAPs connected to detoxification and digestion were chosen for RT-qPCR to validate the proteomic results (**Supplementary Table 1**; **Figure 5**). There was adequate agreement between the RT-qPCR and proteomic data regarding the expression patterns of the twenty selected DAPs, even if some differences were not significant in the RT-qPCR data. The outcomes demonstrated the robustness of the proteomic findings in the current investigation. CYC, GST, and EST enzyme activities were analyzed in the male and female guts of callow and sclerotized beetles (**Figure 6**). Different CYC, GST, and EST enzyme concentrations successfully converted substrate in a concentration-dependent manner (**Supplementary Figure 3**). The activity of CYC and GST-enzyme data was positively correlated with proteomic results, whereas EST-enzymatic data was not fully correlated and demanded further investigation.

### Gene ontology enrichment analysis

**Figure 7:**
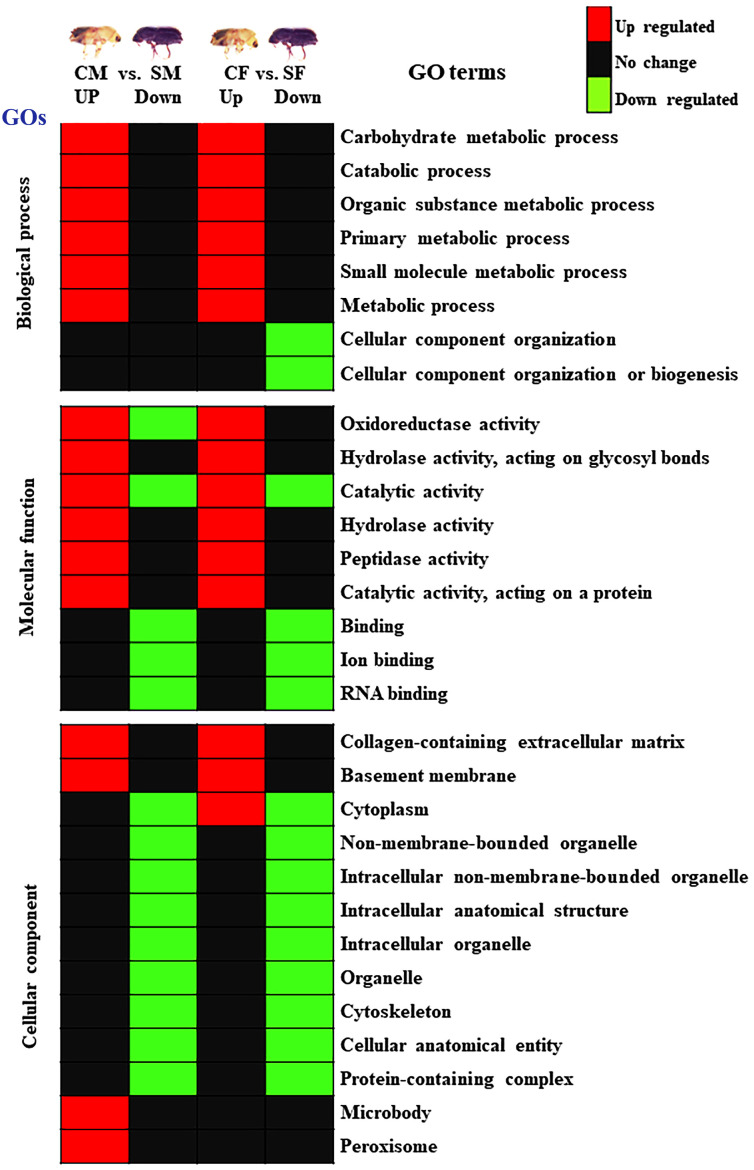
Summary of GO enrichment analysis. Red colour indicates upregulation (>2.0 fold), green colour indicates downregulation (<-2.0 fold), and black indicates no change. CM, callow male; CF, callow female; SM, sclerotized male; SF, sclerotized female.

The protein sequences were searched against the NCBI, InterPro database using Blast2GO, and the resulting biological process, cellular components, and molecular function of the proteins were examined (**Figure 7**; **Supplementary Figure 2**). Most proteins have at least one GO term annotated (**Supplementary Figure 2**). The differentially abundant proteins were separated into two categories to determine which GO term was more prevalent in males or females of both callow and sclerotized *I. typographus*: 1) CF vs. SF and 2) CM vs. SM. The comparison proteins between CF and SF were primarily distributed in the following processes: binding, ion binding, catalytic activity, cellular anatomical entity, intracellular anatomical structure, organelle, intracellular organelle, hydrolase activity, metabolic process, primary metabolic process, organic substance metabolic process, and carbohydrate metabolic process (**Figure 7**; **Supplementary Figure 2**). Besides, the comparison proteins between CM and SM had similar results. However, compared to CF vs. SF, the percentage of each GO term in CM vs. SM was lower.

### DAPs related to metabolisms, detoxification, defence, digestion, cellular signalling, and growth

**Figure 8:**
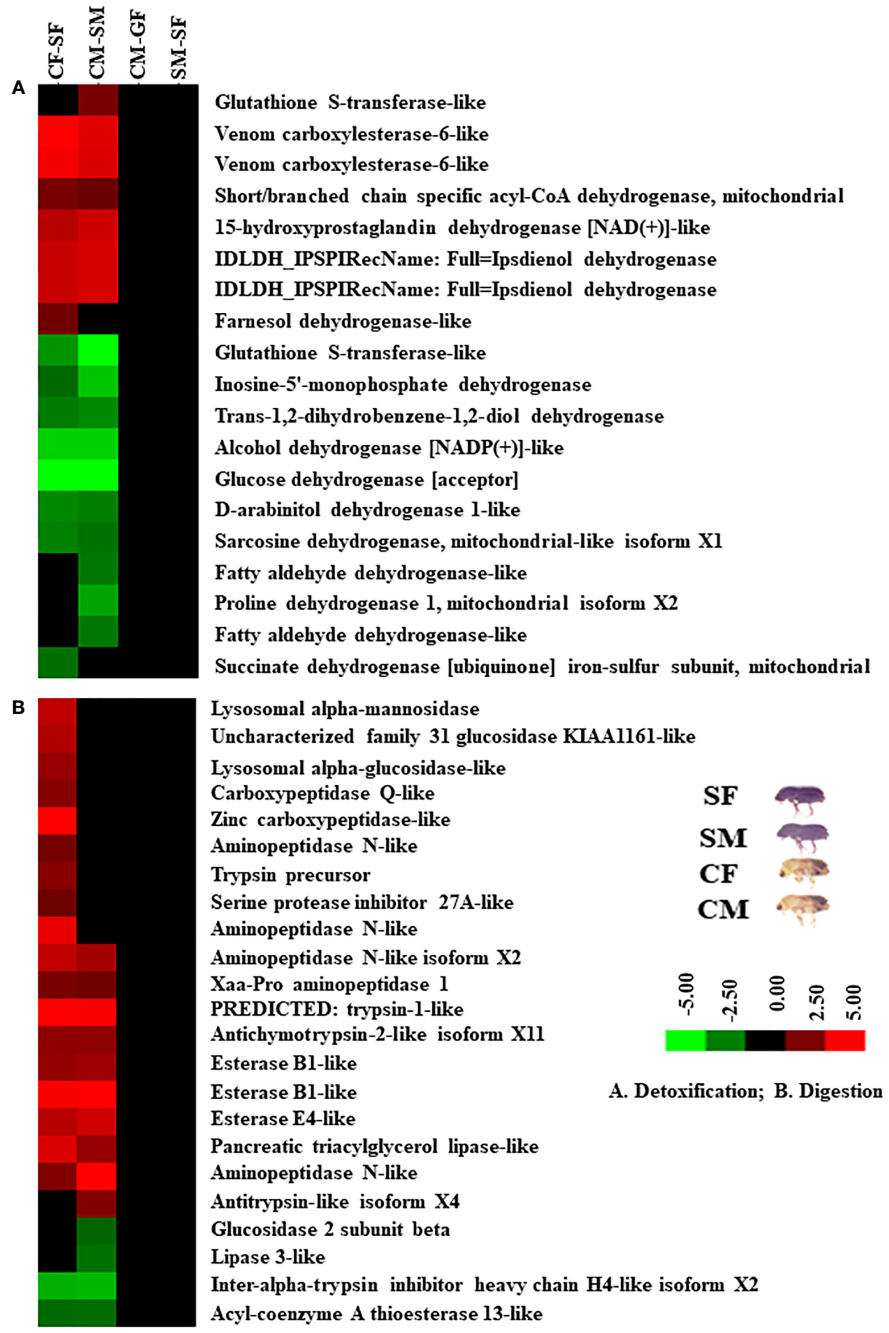
Cluster analysis of DAPs among gut samples of males and females of callow and sclerotized beetles. **(A)** Proteins related to detoxification; **(B)** Proteins related to digestion. Red colour indicates upregulation (>2.0 fold), green colour indicates downregulation (<-2.0 fold), and black indicates no change as compared to respective controls. CM, callow male; CF, callow female; SM, sclerotized male; SF, sclerotized female.

**Figure 9:**
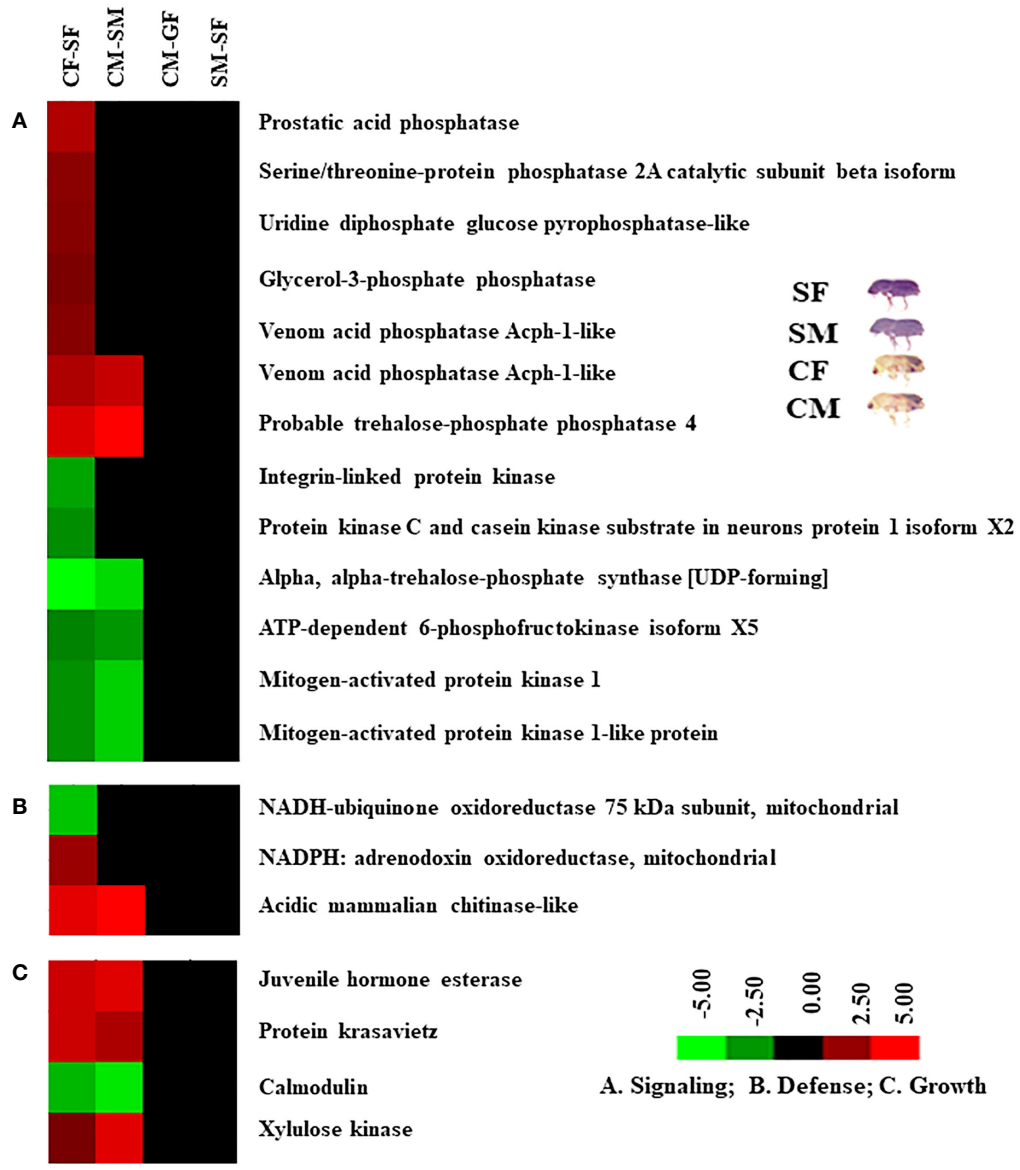
Cluster analysis of DAPs among gut samples of males and females of callow and sclerotized beetles. **(A)** Proteins related to signalling; **(B)** Proteins related to defence; **(C)** Proteins related to growth. Red colour indicates upregulation (>2.0 fold), green colour indicates downregulation (<-2.0 fold), and black indicates no change as compared to respective controls. CM, callow male; CF, callow female; SM, sclerotized male; SF, sclerotized female; DAPs, differentially abundant proteins.

Based on protein expression and analysis, we identified and divided DAP-associated metabolisms into detoxification, digestion, signalling, and growth in the male and female guts of callow and sclerotized beetles (**Figures 8**, **9**). The detoxification and defence-related proteins like venom carboxylesterase-6-like, short/branched chain specific acyl-CoA dehydrogenase, 15-hydroxyprostaglandin dehydrogenase [NAD(+)]-like, ipsdienol dehydrogenase, farnesol dehydrogenase-like are up-regulated and inosine-5’-monophosphate dehydrogenase, trans-1,2-dihydrobenzene-1,2-diol dehydrogenase, alcohol dehydrogenase [NADP(+)]-like, glucose dehydrogenase [acceptor], D-arabinitol dehydrogenase 1-like, sarcosine dehydrogenase, fatty aldehyde dehydrogenase-like, proline dehydrogenase 1, succinate dehydrogenase [ubiquinone] iron-sulfur subunit were down-regulated, whereas glutathione S-transferase-like was differentially regulated in callow compared to sclerotized beetles (**Figure 8A**). The digestion-related proteins like lysosomal alpha-mannosidase, lysosomal alpha-glucosidase-like, carboxypeptidase Q-like, zinc carboxypeptidase-like, aminopeptidase N-like, trypsin precursor, serine protease inhibitor 27A-like, Xaa-Pro aminopeptidase 1, trypsin-1-like, antichymotrypsin-2-like isoform XII, esterase B1-like, esterase E4-like, pancreatic triacylglycerol lipase-like, and antitrypsin-like isoform X4 were up-regulated. Glucosidase 2 subunit beta, lipase 3-like, inter-alpha-trypsin inhibitor heavy chain H4-like isoform X2, and acyl-coenzyme A thioesterase 13-like were down-regulated in callow compared to sclerotized beetles (**Figure 8B**). The signalling-related proteins like prostatic acid phosphatase, serine/threonine-protein phosphatase 2A catalytic subunit beta isoform, uridine diphosphate glucose pyrophosphatase-like, glycerol-3-phosphate phosphatase, venom acid phosphatase Acph-1-like, and trehalose-phosphatase phosphatase 4 were up-regulated. In contrast, integrin-linked protein kinase, alpha, alpha-trehalose-phosphate synthase (UDP-forming), ATP-dependent 6-phosphofructokinase isoform X5, and mitogen-activated protein kinase 1 were down-regulated in callow compared to sclerotized beetles (**Figure 9A**). The defence-related proteins like NADPH: adrenodoxin oxidoreductase and acidic mammalian chitinase-like were up-regulated, whereas NADH-ubiquinone oxidoreductase 75 kDa subunit is down-regulated in callow compared to sclerotized beetles (**Figure 9B**). The growth-related proteins like juvenile hormone esterase, protein krasavietz, and xylulose kinase were up-regulated, whereas calmodulin is down-regulated in callow compared to sclerotized beetles (**Figure 9C**).

**Figure 10:**
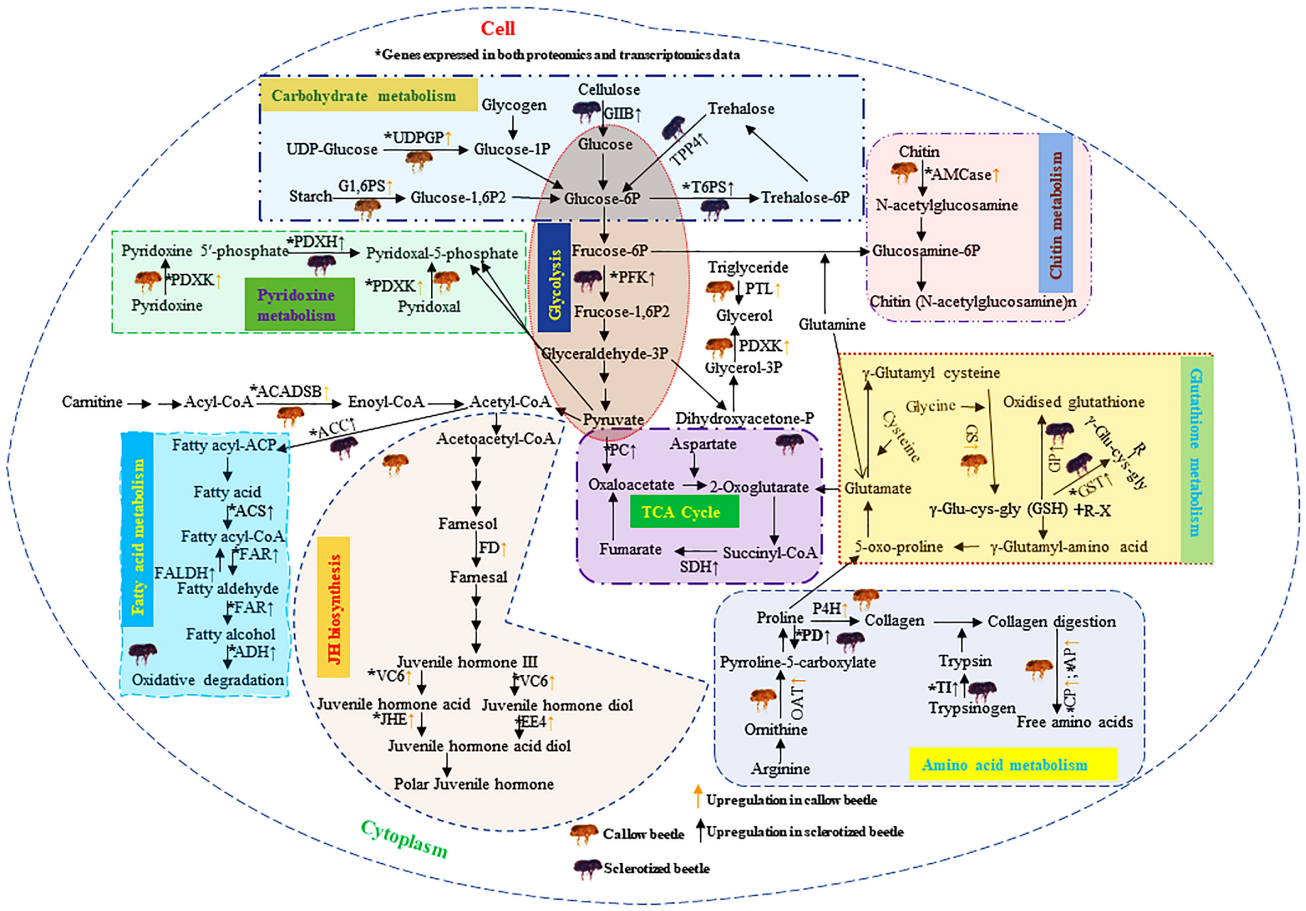
Diagrammatic representation of DAPs analysis in the callow and sclerotized males and females compared to respective controls. The yellow (↑) and black (↑) arrows represent the upregulated genes (FDR corrected p-value<0.05) in callow and sclerotized beetles, respectively. The upregulated protein-coding enzymes in callow beetles are mainly related to JH biosynthesis, gluconeogenesis, amino acid, and pyridoxine metabolism. The overexpression of these proteins may aid in converting callow to sclerotized beetles. The upregulated proteins in sclerotized beetles are mostly connected to carbohydrates, fatty acid, chitin, trypsin, and glutathione metabolisms. It is possible that sclerotized beetles are the pioneer for starting a new colony and may aid energy production and detoxification of host allelochemicals. Carbohydrate metabolism: Glucosidase 2 subunit beta (GIIB), glucose 1,6-bisphosphate synthase (G1,6PS), uridine diphosphate glucose pyrophosphatase-like (UDPGP), alpha,alpha-trehalose-phosphate synthase [UDP-forming] (T6PS), probable trehalose-phosphate phosphatase 4 (TPP4), ATP-dependent 6-phosphofructokinase isoform X5 (PFK), pyruvate carboxylase, mitochondrial (PC), and succinate dehydrogenase [ubiquinone] iron-sulfur subunit, mitochondrial (SDH). Fatty acid metabolism: Acetyl-CoA carboxylase (ACC), acyl-CoA synthetase family member 3 (ACS), mitochondrial-like fatty acyl-CoA reductase wat-like isoform X2 (FAR), Fatty aldehyde dehydrogenase-like (FALDH), fatty acyl-CoA reductase wat-like isoform X2 (FAR), Alcohol dehydrogenase [NADP(+)]-like (ADH), Short/branched chain specific acyl-CoA dehydrogenase, mitochondrial (ACADSB), glycerol-3-phosphate phosphatase (G3PP), and pancreatic triacylglycerol lipase-like (PTL). JH biosynthesis: Farnesol dehydrogenase-like (FD), Venom carboxylesterase-6-like (VC6), Juvenile hormone esterase (JHE), and esterase E4-like (EE4). Glutathione metabolism: Glutathione synthetase-like isoform X2 (GS), probable phospholipid hydroperoxide glutathione peroxidase isoform X3 (GP), Glutathione S-transferase (GST). Amino acid metabolism: Ornithine aminotransferase, mitochondrial (OAT), proline dehydrogenase 1, mitochondrial isoform X2 (PD), prolyl 4-hydroxylase subunit alpha-1-like isoform X1 (P4H), inter-alpha-trypsin inhibitor heavy chain H4-like isoform X2 (TI), carboxypeptidase Q-like (CP), and aminopeptidase N-like (AP). Chitin metabolism: Acidic mammalian chitinase-like (AMCase). Pyridoxin metabolism: Pyridoxal kinase (PDXK) and Pyridoxine-5’-phosphate oxidase (PDXH), Glutathione (GSH), Xenobiotics (R-X). DAPs: differentially abundant proteins.

The upregulated protein-coding enzymes in callow beetles are mainly related to JH biosynthesis (farnesol dehydrogenase-like, venom carboxylesterase-6-like, juvenile hormone esterase, and esterase E4-like), amino acid (ornithine aminotransferase, proline dehydrogenase 1, prolyl 4-hydroxylase subunit alpha-1-like isoform X1, carboxypeptidase Q-like, and aminopeptidase N-like), and pyridoxine metabolism (pyridoxal kinase and pyridoxine-5’-phosphate oxidase) (**Figure 10**). The overexpression of these proteins may aid in converting callow to sclerotized beetles. The upregulated proteins in sclerotized beetles are mostly connected to carbohydrates (glucosidase 2 subunit beta, glucose 1,6-bisphosphate synthase, uridine diphosphate glucose pyrophosphatase-like, alpha,alpha-trehalose-phosphate synthase [UDP-forming], probable trehalose-phosphate phosphatase 4, ATP-dependent 6-phosphofructokinase isoform X5, pyruvate carboxylase, and succinate dehydrogenase [ubiquinone] iron-sulfur subunit), fatty acid (acetyl-CoA carboxylase, acyl-CoA synthetase family member 3, mitochondrial-like fatty acyl-CoA reductase wat-like isoform X2, fatty aldehyde dehydrogenase-like, fatty acyl-CoA reductase wat-like isoform X2, alcohol dehydrogenase [NADP(+)]-like, short/branched chain specific acyl-CoA dehydrogenase, glycerol-3-phosphate phosphatase, and pancreatic triacylglycerol lipase-like), chitin (acidic mammalian chitinase-like), trypsin (inter-alpha-trypsin inhibitor heavy chain H4-like isoform X2), and glutathione metabolisms (glutathione synthetase-like isoform X2, probable phospholipid hydroperoxide glutathione peroxidase isoform X3, glutathione S-transferase) (**Figure 10**).

## Discussion

We find minimal differences in sex-specific protein expression between the two adult beetle stages. Instead, the protein expression difference between callow and sclerotized beetle gut tissues is much more noticeable, supporting their different ecological roles and life-stage-specific requirements. The differential protein expression between callow and sclerotized beetles may be a sign of different amounts of the feeding of host tissues. Callow beetles feed rigorously to attain the mature stage, hence investing more resources in the digestion and detoxification of food to prepare themselves for new host finding. Alternatively, sclerotized beetles stored enough energy for flight and host finding for colonization.

### Functional prediction of DAPs: reflecting key physiological processes in beetle adult stages

Ecdysteroid and sesquiterpenoid pathways regulate embryogenesis, developmental change, and growth in insects (Adhitama et al., 2020). In this study, GO terms related to the juvenile hormone catabolic process, juvenile hormone metabolic process, sesquiterpenoid catabolic process, and sesquiterpenoid metabolic process under biological processes were upregulated in the callow males and females (**Supplementary Figure 2**), suggesting that the proteins may help callow beetles converting to become fully mature sclerotized beetles. Genetic, molecular, and cytological investigations on *Drosophila* have provided information on the precise roles played by the cytoskeleton during oogenesis (Cooley and Theurkauf, 1994). In this study, the biological process GO terms related to cellular component organization and cellular component biogenesis were upregulated in sclerotized females, suggesting that these proteins may have a role in cytoskeleton development and reproduction (**Figure 7**).

The RNA helicases Hlc, hel, eIF-4a, vasa, and mle have all been discovered in *Drosophila melanogaster.* These helicases appear to be engaged in various cellular functions, including the development of the embryonic body plan, the association of salivary gland chromosomes and embryonic cell nuclei, and male viability (Lasko and Ashburner, 1988; Kuroda et al., 1991; Dorn et al., 1993; de Couet et al., 1995; Eberl et al., 1997). RNA interference (RNAi)-mediated suppression of the Na+/K+-ATPase gene in the migrating locust results in patency losses, inhibiting Vg absorption, egg maturation and hindering ovarian development (Jing et al., 2018). The molecular function GO terms related to ATP-dependent activity, cytoskeletal protein binding, helicase activity, and pyrophosphatase activity were downregulated in callow females and upregulated in sclerotized females (**Supplementary Figure 2**), suggesting that they may help sclerotized female beetles for patency formation, Vg absorption, egg maturation, and ovarian development.

Collagen is ubiquitous throughout the animal kingdom, comprising some 28 diverse molecules forming the extracellular matrix within organisms (Sutherland et al., 2013; Halliwell and Gutteridge, 2015). In this study, the cellular component GO terms related to collagen-containing extracellular matrix and basement membrane proteins are upregulated (**Figure 7**) in male and female callow beetles, suggesting they may be involved in beetle metamorphosis.

### DAPs contributed to beetle metabolisms, detoxification, and defence

Plants produce various allelochemicals to defend themselves against herbivory (Johnson, 2011; Saxena et al., 2023; Yadav et al., 2023). The resin in conifers is the essential component of chemical defence against the attacks of bark beetles and can inhibit beetle invasion, slow the invasion speed, and even repel the beetles (Erbilgin et al., 2003; Erbilgin et al., 2007). Moreover, the monoterpene in the resin is entomotoxic and causes debility or death through the respiratory system (Smith, 1965; Fernanda López et al., 2011). Our results are consistent with previous studies comparing transcriptional responses between generalist and specialist species (phytophagous insects) and with notable variations in the expression of genes associated with either direct or indirect xenobiotic detoxification between the two behaviours (Ali and Agrawal, 2012; Roy et al., 2016; Schweizer et al., 2017). The gut transcriptome of two *Dendroctonus* bark beetle species (*D. rhizophagus* and *D. valens*) stimulated by the same kairomones reveals molecular variations in detoxification pathways, such as up-regulated unigenes in *D. rhizophagus* (P450s and some COEs) that were different from those in *D. valens* (COEs, GSTs, and ABC transporters) (Torres-Banda et al., 2022). The glutathione S-transferases (GSTs) are detoxification enzymes that conjugate toxins with glutathione and play a role in excretion. To better understand the interaction between monoterpenes and beetles, sixteen full-length sequences were identified from four GST categories (delta, epsilon, sigma, and theta) and compared at four developmental stages (larvae, pupae, teneral adults, and adults), three different tissues (antennae, gut, and reproductive organs), and under various levels of terpenoid stimuli during feeding in *D. armandi* (Gao et al., 2020). While the numbers of cytosolic GSTs were similar among the beetle species *D. valens*, *D. rhizophagus*, *D. ponderosae*, *L. decemlineata*, and *A. glabripennis* but observed noteworthy differences in the Sigma, Delta, and Epsilon classes (Torres-Banda et al., 2022). The Zeta GSTs have been linked to the breakdown of tyrosine and phenylalanine following the completion of the cuticle sclerotization process, as well as being connected to the detoxification of xenobiotics containing chloride compounds (BOARD et al., 1997; Yamamoto et al., 2009). The existence of Omega GSTs in *Dendroctonus species*, *Apis cerana*, and *Rhopalosiphum padi* implies that these GSTs could have a significant function in helping these species adapt to their toxic-laden environment (Zhang et al., 2013; Dai et al., 2016; Balakrishnan et al., 2018). Furthermore, the presence of Sigma GSTs in *Dendroctonus* species points to a potential role for these proteins in oxidative stress defence through conjugation activity with lipid peroxidation products and carbaryl insecticides, which may have enabled these bark beetles to evolve survival strategies in toxin-rich subcortical environments (Fernanda López et al., 2011; Krokene, 2015; Gao et al., 2020; Dai et al., 2021). As bark beetles thrive on conifer host tissues in all life stages except egg and pupae, the Delta, Epsilon, and unclassified classes of GSTs are probably associated with beetle resistance to host secondary metabolites and terpenes (Liu et al., 2021). In the current study, the glutathione S-transferase genes are differentially regulated between callow and sclerotized males (**Figures 6**, **8A**). Sclerotized beetles are attacking and drilling the bark for colony establishment; hence, they may require more GSTs to deal with the initial spruce defence response. However, the major spruce monoterpenes lack the electrophilic sites to react directly with glutathione. Hence, we speculate that the fungal symbionts of bark beetle species metabolize spruce resin monoterpenes and alter the profile of spruce bark volatiles by converting the major monoterpenes into an attractive blend of oxygenated derivatives, which might act as the GST substrate (Kandasamy et al., 2023). Alternatively, the monoterpenes stimulate ROS production, causing the production of oxidized metabolites, such as hydroperoxides, which could also be GST substrates. Furthermore, toxic piperidine alkaloids of spruce can also be the substrate for GST due to the presence of electrophilic sites (Tawara et al., 1993; Stermitz et al., 2000). However, such possibilities demand further experimental corroboration.

Members of the carboxylesterases (COEs) clades A and C are found in the dietary and detoxification functions class, which is broadly represented in all insects, whereas clade A is unique and diverse in the phylogeny of bark beetles and is primarily involved in the metabolism of xenobiotics (Lü et al., 2015; Sood et al., 2016). The up-regulation of COEs in *D. valens*, *D. armandi*, and *D. ponderosae* following exposure to the kairomone blend and their phloem feeding demonstrates the essential role of these COEs in terpene metabolism (Robert et al., 2013; Dai et al., 2019; Dai et al., 2021). A recent study identified the potential insecticide resistance marker in the venom carboxylesterase-6-like gene through genomic-level comparison of the current field and 1976 year collected *Bemisia tabaci* (Gennadius) lines, suggests that venom carboxylesterase-6-like gene play a role in the insecticide resistance mechanism (Wang et al., 2022). In *Aphidius gifuensis*, the antennae- and sex-specific transcriptome study, phylogenic analysis, and tissue-expression patterns identified venom carboxylesterase-6-like as a critical ODE involved in the chemical breakdown (Kang et al., 2021). In the present study, compared to the sclerotized beetles, the venom carboxylesterase-6-like gene shows higher expression (3.57 to 5.5 folds) in both males and females of callow beetles (**Figure 8A**), suggesting its putative involvement in the detoxification of plant chemicals during maturation feeding.

Alcohol dehydrogenases (ADHs) are required for xenobiotics detoxification, especially for the oxidation of permethrin (Choi et al., 2002). In *S. zeamais*, the upregulation of ADHs shows improved critical substrate conservation in the carbohydrate metabolism, consequently increasing the xenobiotics metabolism (Huang et al., 2019). Prior research has demonstrated the presence of ethanol in both xylem and phloem tissues, with its concentration increasing in plant tissues during bark beetle attacks (Kelsey et al., 2014; Lehenberger et al., 2021). High concentrations of ethanol are hazardous to bark beetles, and alcohol dehydrogenase is responsible for metabolizing the majority of the ethanol absorbed as part of the diet, and its action may help to quickly eliminate the ethanol from the bark beetle hemolymph (Dettner, 2023). As ethanol is also toxic to pathogens or fungal antagonists of bark beetle nutritional fungal mutualists, thriving on the eatnol-rich host substrate using ADH may give them a selective survival advantage (Lehenberger et al., 2021). Compared to the callow *I. typographus*, the alcohol dehydrogenase [NADP(+)]-like gene has higher expression (4 fold up) in both male and female sclerotized beetles (**Figure 8A**), implying its requirement while establishing a new colony and making the galleries for oviposition. The dietary alkaloid-fed honeybee proteomic and metabolomic analysis showed upregulation of succinate dehydrogenase and glucose dehydrogenase, rate-limiting enzymes in the TCA cycle, and pentose phosphate pathway, signifying that they may be involved in NADH and NADPH production required for the P450 functioning and cellular redox state maintenance (Esther et al., 2017). In *I. typographus*, glucose dehydrogenase [acceptor] (-7 fold) and succinate dehydrogenase [ubiquinone] (-2.22 fold) were downregulated in callow males compared to the sclerotized males (**Figure 8A**), implying that they may require continuous energy production during new host finding. In male *I. pini* beetles, the ipsdienol dehydrogenase (IDOLDH) is an essential enzyme in pheromone biosynthesis that oxidizes explicitly (−)-ipsdienol and (−)-ipsenol to ipsdienone and ipsenone, respectively, and is thought to “tune” ipsdienol enantiomeric ratios (Figueroa-Teran et al., 2012; Figueroa-Teran et al., 2016). Ipsdienol dehydrogenase (IDLDH) is upregulated (3.87 to 4.19 folds) in the male and female callow beetles compared to sclerotized ones (**Figure 8A**), suggesting that host feeding (i.e., including alpha-pinene) induces pheromone production for future chemical communication during host colonization in *I. typographus*.

Insect life, moulting, and development depend on at least some chitinases belonging to various families (Arakane and Muthukrishnan, 2010). Also, bacterial associates of bark beetles may be involved in the production of metabolites such as chitinases to serve as a protective barrier against pathogens, as well as boosting the metabolic capacity to fulfil its nutritional requirements through the hydrolysis of complex sugar polymers that are typically indigestible into simpler, more readily assimilated sugars (García‐Fraile, 2018; Chakraborty et al., 2020; Chakraborty et al., 2023; Peral-Aranega et al., 2023). In this present study, the acidic mammalian chitinase-like gene was upregulated (4.16 to 5.36 folds) in males and females of callow beetles compared to sclerotized beetles (**Figure 9B**), referring to its necessity during the transition to sclerotized beetles. In *Drosophila melanogaster*, adrenodoxin reductase (ADXR) is encoded by the nuclear gene dare and plays a crucial role in ecdysteroid production when mutant larvae are fed the insect steroid hormone 20-hydroxyecdysone, null mutants of the dare gene experience developmental arrest. The NADPH: adrenodoxin oxidoreductase (mitochondrial) gene was found to be significantly expressed (2.54 fold) in the callow females in the current study (**Figure 9B**), indicating that it may aid in the storage of ecdysteroids and their use in the conversion of callow females to sclerotized females in addition to their putative role in the detoxification mechanism (Freeman et al., 1999). Further functional studies may delineate the functional role of such crucial enzymes in the bark beetle life cycle.

### DAPs involved in beetle digestion

Insect digestive proteins are mainly produced and secreted into the lumen after being directed by midgut epithelial cells, and through the lumen, food particles pass (Hegedus et al., 2009; Napoleão et al., 2019). In lepidopteran insects, aminopeptidase N (APN) isoforms play a role in the mode of action of *Bacillus thuringiensis* insecticidal *Cry* proteins (Crava et al., 2013). The Aminopeptidase-N is also found in the midgut of coleopterans and is a protein that binds to *Cry3Aa* toxins (Guo et al., 2020). In this study, aminopeptidases (aminopeptidase N-like, aminopeptidase N-like isoform X2, and xaa-Pro aminopeptidase 1) are significantly upregulated (2.22 to 5.35 folds) in male and female callow beetles compared to the sclerotized beetles (**Figure 8B**), suggesting its involvement in digestion of plant materials during the rigorous maturation feeding. Trypsin-like enzymes are involved in moulting, tissue remodelling, innate immunity, diapause, fertilization, female postmating behaviour regulation, and activation of enzyme precursors of trypsin, chymotrypsin, chitinase, phenoloxidase, and *Bt*-toxin degradation (Friedländer et al., 2001; Royer et al., 2002; Wagner et al., 2002; Kanost et al., 2004; Chen et al., 2005; Peng et al., 2005; Wei et al., 2007; Liu et al., 2009; Parde et al., 2012). In the current study, antichymotrypsin-2-like isoform X11 and trypsin-1-like genes are upregulated (2.78 to 5.01 folds) in both male and female callow beetles (**Figure 8B**), implying their involvement in the spruce tissue digestion. Trypsin precursor and antitrypsin-like isoform X4 were upregulated (2.67 and 2.52 folds) in callow females and males, suggesting they may contribute to development, reproductive regulations, and tissue remodelling (**Figure 8B**).

An RNAi study in *Nilaparvata lugens* recently showed that injected dsRNA against inter-alpha-trypsin inhibitor heavy chain 4 (ITIH4) reduced survival, egg production, egg hatching, and delayed ovarian development. These results indicate that ITIH4 plays a vital role in development and reproduction (Ji et al., 2022). In *I. typographus*, inter-alpha-trypsin inhibitor heavy chain H4-like isoform X2 was downregulated in both male (-3.56 fold) and female (-3.43 fold) callow beetles compared to the sclerotized ones, suggesting that it may involve in the development and reproduction in sclerotized beetles (**Figure 8B**). In *Culex pipiens quinquefasciatus*, high expression of the esterase B1 gene shows insecticide resistance (Mouches et al., 1990). Esterase B1-like shows high expression (2.90 to 5.51 folds) in both males and females of callow beetles compared to the sclerotized beetles, indicating their involvement in coping with allelochemicals during active host feeding (**Figure 8B**). The pancreatic triglyceride lipase (PTL) gene knockdown brown planthopper (*Nilaparvata lugens*), insects fed to insect-resistant rice varieties showed less food intake, lipid content, survival rate, and fecundity (Yuan et al., 2020). The pancreatic triacylglycerol lipase-like gene is upregulated in both male (2.93 fold) and female (4.28 fold) callow beetles to aid the digestion of food materials and reproductive development (**Figure 8B**). Carboxypeptidase Q may play an essential role in the hydrolysis of the circulating peptides and catalyzes unsuitable terminals into amino acids (Gatehouse, 2013). The zinc carboxypeptidase belongs to the B type and prepared COOH-terminal residue. Zinc carboxypeptidase expression rapidly increased in teneral flies after 1-hour post-bloodmeal feeding compared to unfed (Yan et al., 2002). In *I. typographus*, carboxypeptidase Q-like, and zinc carboxypeptidase-like genes are upregulated (2.64 and 5.35 folds) in female callow beetles compared to sclerotized female beetles, suggesting their putative requirement for initiating the ovary development for egg formation during the callow to sclerotized beetle transition (**Figure 8B**). However, such possibilities need functional endorsements.

In *Drosophila*, serine protease inhibitor 27A regulates the melanization cascade by explicitly inhibiting the terminal protease prophenoloxidase activating enzyme. Serine protease inhibitor 27A-like gene was upregulated (2.19 fold) in female callow compared to the sclerotized female beetles, endorsing their similar role in melanization in beetles (**Figure 8B**). The xylulose kinase phosphorylates the D-xylulose to produce D-xylulose-5-phosphate and is essential in regulating glucose metabolism and lipogenesis (Bunker et al., 2013). In the present study, the xylulose kinase is upregulated (4.06 and 2.01 folds) in both callow males and females to support the digestion of plant carbohydrates during maturation feeding (**Figure 9C**).

### DAPs involved in beetle cellular signalling

RNAi studies in the Cabbage Beetle (*Colaphellus bowringi*) revealed that the MAPK signalling pathway is essential for insect female reproductive regulation (Huang et al., 2022). Mitogen-activated protein kinase 1 (MAPK 1), also known as an extracellular signal-regulated kinase 2 (ERK2) or MAPK p38, is involved in insect defence against Bt Cry toxins (Cancino-Rodezno et al., 2010). In this study, the mitogen-activated protein kinase 1 and mitogen-activated protein kinase 1-like protein expression is higher in sclerotized male and female beetles than the callow beetles, plausibly aiding in mitigating challenges by pathogens during establishing a new colony (**Figure 9A**). The trehalose-phosphate phosphatase 4 is involved in the pathway trehalose biosynthesis, which removes the phosphate from trehalose 6-phosphate to produce free trehalose. The alpha, alpha-trehalose-phosphate synthase [UDP-forming], also known as trehalose phosphate-UDP glucosyltransferase, is a catalytic subunit of the trehalose synthase complex that catalyzes the production of trehalose from glucose-6-phosphate and UDP-alpha-D-glucose in a two-step process. In *I. typographus*, alpha-trehalose-phosphate synthase [UDP-forming] and ATP-dependent 6-phosphofructokinase isoform X5 were upregulated (3.87 to 4.97 folds, and 2.48 and 2.16 folds) in male and female sclerotized beetles (**Figure 9A**). These results suggest that sclerotized beetles need energy for flying, especially sclerotized male beetles, which are the pioneers in establishing the colony, and these genes are mainly involved in trehalose metabolism and provide energy for flying.

### DAPs involved in beetle growth

Juvenile hormone (JH) is a factor produced by the corpora allata glands that prevent immature insects from moulting into adults, and rates of its biosynthesis and degradation regulate the JH (Kamita et al., 2003). In lepidopteran larvae, inhibition of Juvenile hormone esterases (JHE) reduces the rate of JH degradation, leading to abnormally large larvae and delayed pupation (Hammock et al., 1990). In *I. pini*, an analysis of expressed sequence tags (ESTs) revealed the upregulation of seven mevalonate genes in midgut tissues, indicating their putative responsiveness to JHIII (Eigenheer et al., 2003). In the present study, the juvenile hormone esterase is upregulated (4.0 and 3.57 folds) in male and female callow beetles compared to the sclerotize group, suggesting their putative involvement in the metamorphic process of callow beetles (**Figure 9C**). Calmodulin (CaM) is one of the most evolutionarily conserved Ca^2+^ sensors in organisms and plays a role in the stress response of insects to the diamide insecticide cyantraniliprole (Guo et al., 2021). In *S. frugiperda*, calmodulin plays a role in the midgut microapocrine secretory process of digestive enzymes (Ferreira et al., 2007; Silva et al., 2013). Calmodulin was upregulated (2.88 and 3.53 folds) in the male and female callow beetles compared to the sclerotized group, plausibly to induce the secretion of digestive enzymes for conifer food digestion during maturation feeding (**Figure 9C**).

### DAPs and DETs correlation: unveiling conserved responses

Through our investigation between gut proteome and transcriptome of both callow and sclerotized beetles, we identified numerous DAPs specific to each adult stage, and these findings illuminate the multifaceted roles of these proteins and how they contribute to the life histories, ecological strategies, and adaptive physiological responses of *I. typographus* in effectively mitigating the toxicity of host allelochemicals. A subset of transcripts and proteins were expressed with the same pattern (**Figure 2**). Most shared proteins are engaged in conserved processes such as gut development, detoxification, digestion, defence, signalling, and growth, respectively. A similar correlation trend was found in the study of the heat stress-accumulated transcriptome and proteome in *Neoseiulus barkeri* (Tian et al., 2020). The ecological relevance of such DAPs or DETs with conserved expression can be an exciting research avenue for better understanding the beetle adaptive physiology and may provide some valuable target genes for management purposes.

## Conclusion

This is the first study investigating the regulatory mechanisms of digestion, detoxification, and growth in callow and sclerotized beetles from the *I. typographus* gut protein expression perspective. DAP analysis, DAP and DET comparison, enzymatic assays, and RT-qPCR validation results indicated that the *I. typographs* gut protein expression pattern played a significant role in digestion, detoxification, and growth in callow or sclerotized stages (**Figure 10**). The current study sheds light on how the fine-tuning of protein expression in the gut is based on the physiological requirements of the beetle adult stages (**Figure 10**). This aspect is not considered before in other bark beetles. In this study, we found that proteins related to JH biosynthesis and chitin metabolism are upregulated in callow beetles, whereas proteins related to fatty acid metabolism and the TCA cycle are upregulated in sclerotized beetles. These differential protein regulation patterns may aid *I. typographus* in fulfilling their life-stage-specific ecological duties (**Figure 10**) and create new avenues for future functional studies. Nevertheless, current data will facilitate further protein-level studies in this beetle or other coleopteran forest pests, creating the possibility of formulating superior forest pest management strategies targeting essential proteins using RNAi with superior delivery methods (i.e., nanoparticle or microbe-mediated delivery) applicable to forestry (Joga et al., 2021; Gupta et al., 2023; Mogilicherla and Roy, 2023; Sandal et al., 2023).

## Data availability statement

The mass spectrometry proteomics data have been deposited to the ProteomeXchange Consortium via the PRIDE [1] partner repository with the dataset identifier PXD035407.

## Ethics statement

The manuscript presents research on animals that do not require ethical approval for their study.

## Author contributions

AR conceptualization. MA, GS sample collection, experimental work. VS, FL proteomics data generation and processing. KM, MA, GS biological interpretation, figure preparation, and first draft wiring. AR and KM prepared the final draft. All authors read and approved the final version of the draft.

## Supplementary material

Supplementary Figure 1 Standardization of total protein isolation and quality checking. **(A)**. Total protein was isolated from six different gut samples, run on the SDS-PAGE gel, and checked the protein quality. **(B)**. After reviewing the protein quality and loading on stacking gel, run up to the separating gel, cut the protein band, and carry on the in-gel digestion process. M. Protein marker; 1 to 6: Biological replicates of gut protein. *Six biological replicates were utilized from each sample for total protein isolation, followed by four high-quality samples for protein sequencing.

Supplementary Figure 2 Bar graphs showing gene ontology classification between four comparisons. Category and functional distribution of the 539 DAPs between four comparisons according to the significant classifications of gene ontology: Biological Process, Molecular Function, and Cellular Components. **(A)**. GF vs SF Upregulated; **(B)**. GF vs SF Downregulated; **(C)**. GM vs SM Upregulated; **(D)**. GM vs SM Downregulated. CM: callow male; CF: callow female; SM: sclerotized male; SF: sclerotized female; DAPs: differentially abundant proteins.

Supplementary Figure 3 CYC **(A)**, GST **(B)**, and EST **(C)**- influence of enzyme source concentration (0.5, 1, 2, and 4 µg) and time course (30min) on reaction rates (ΔAbs (u)/min). **(A)** sclerotized male (SM), **(B)** sclerotized female (SF), **(C)** callow male (CM), and **(D)** callow female (CF). Error bars correspond to standard error (total number of guts tested (n = 48).

Supplementary Excel File 1 Supporting information for differentially abundant proteins in various comparisons.

Supplementary Excel File 2 Supporting information for gene expression at transcriptomics and proteomics level.

Supplementary Table 1 Genes and their primer sequences used for RT-qPCR.
