# Supplementary material for: Comparative gut proteomics study revealing adaptive physiology of Eurasian spruce bark beetle, Ips typographus (Coleoptera: Scolytinae)
Source: Front Plant Sci. 2023 Nov 21;14:1157455. doi: 10.3389/fpls.2023.1157455 (PMC10703158; doi:10.3389/fpls.2023.1157455)
Supplement: Supplementary Figure 1 — Standardization of total protein isolation and quality checking. (A). Total protein was isolated from six different gut samples, run on the SDS-PAGE gel, and checked the protein quality. (B). After reviewing the protein quality and loading on stacking gel, run up to the separating gel, cut the protein band, and carry on the in-gel digestion process. M. Protein marker; 1 to 6: Biological replicates of gut protein. *Six biological replicates were utilized from each sample for total protein isolation, followed by four high-quality samples for protein sequencing. [file DataSheet_1.pdf]

Supplementary figure 1

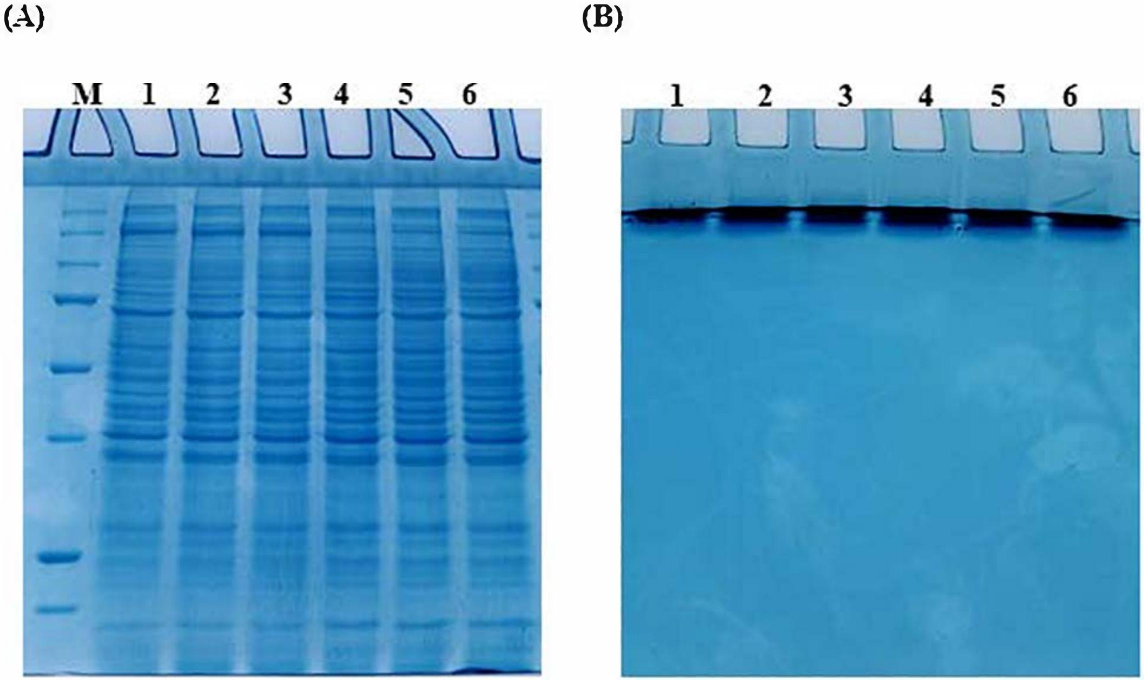

Supplementary figure 2

(A). CF vs. SF Upregulated Enriched Bar Chart

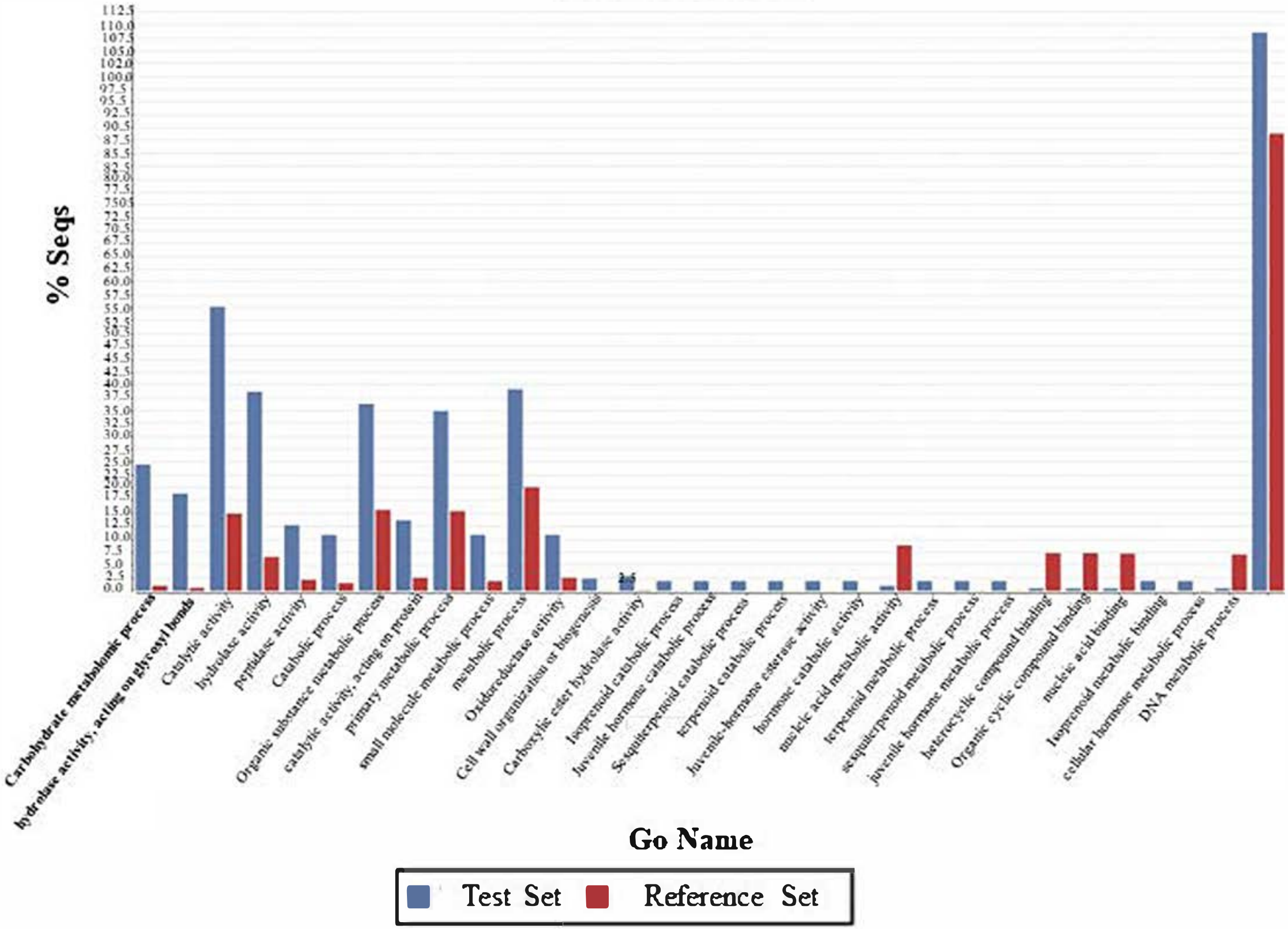

Supplementary figure 2

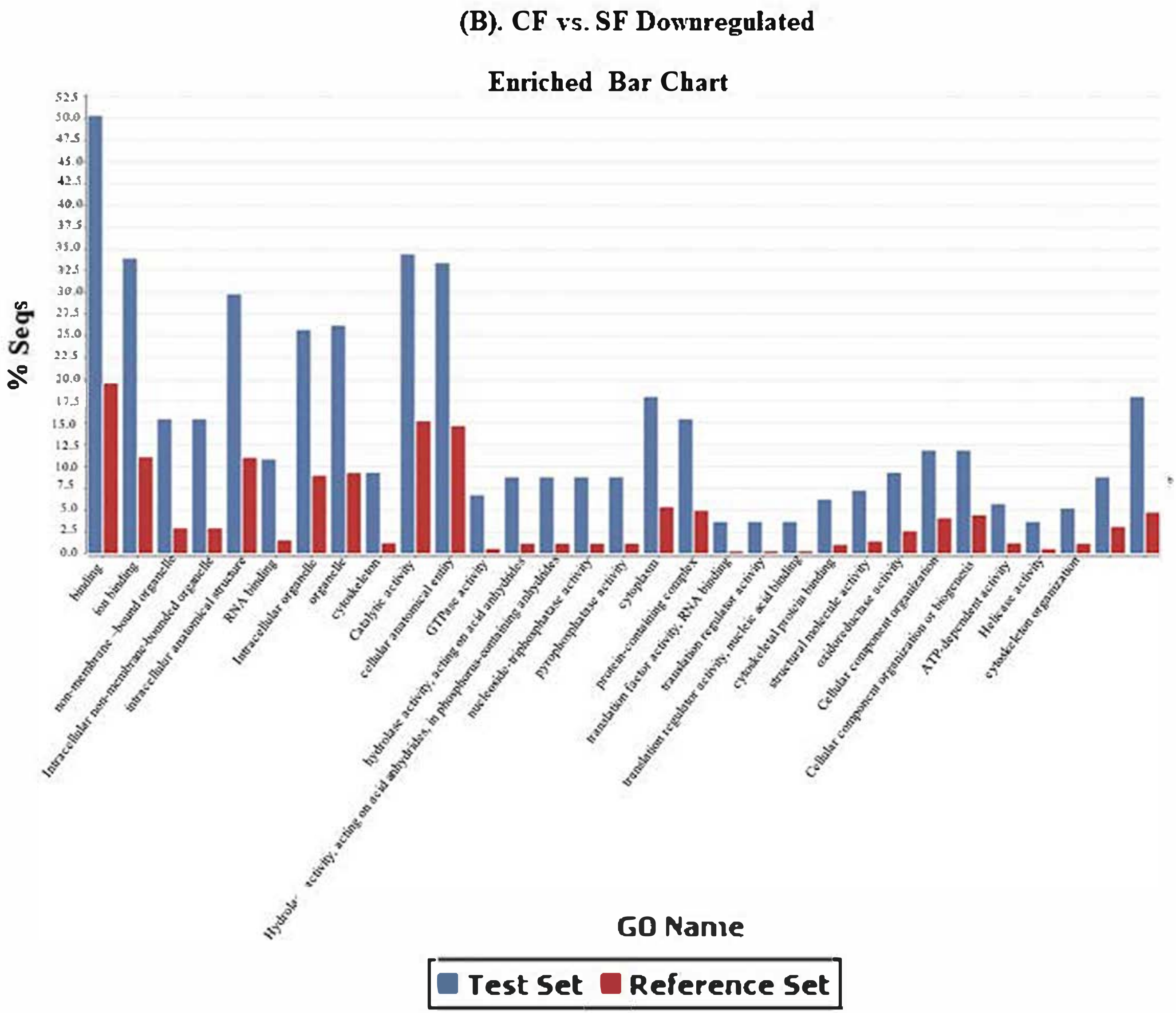

Supplementary figure 2

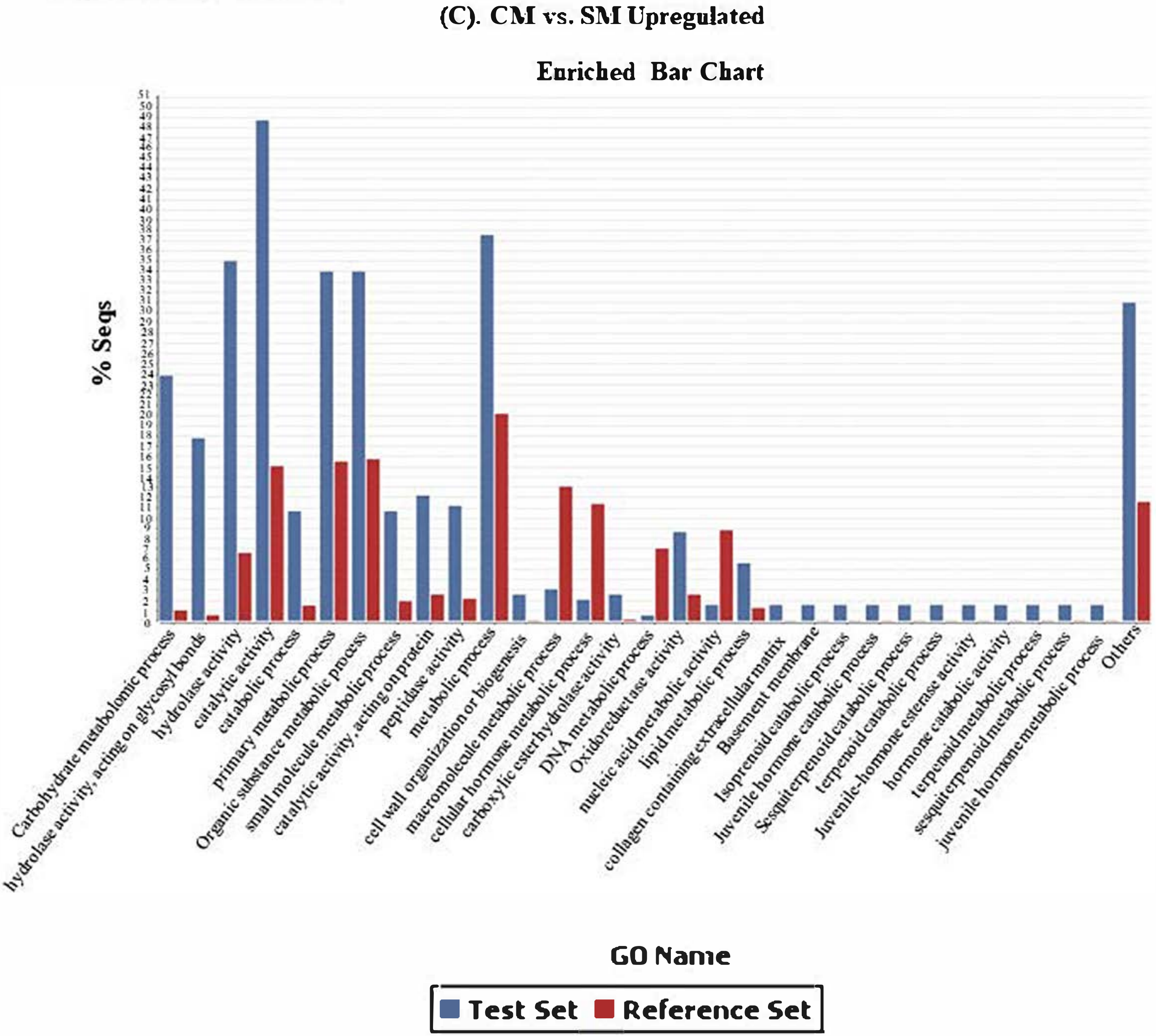

Supplementary figure 2

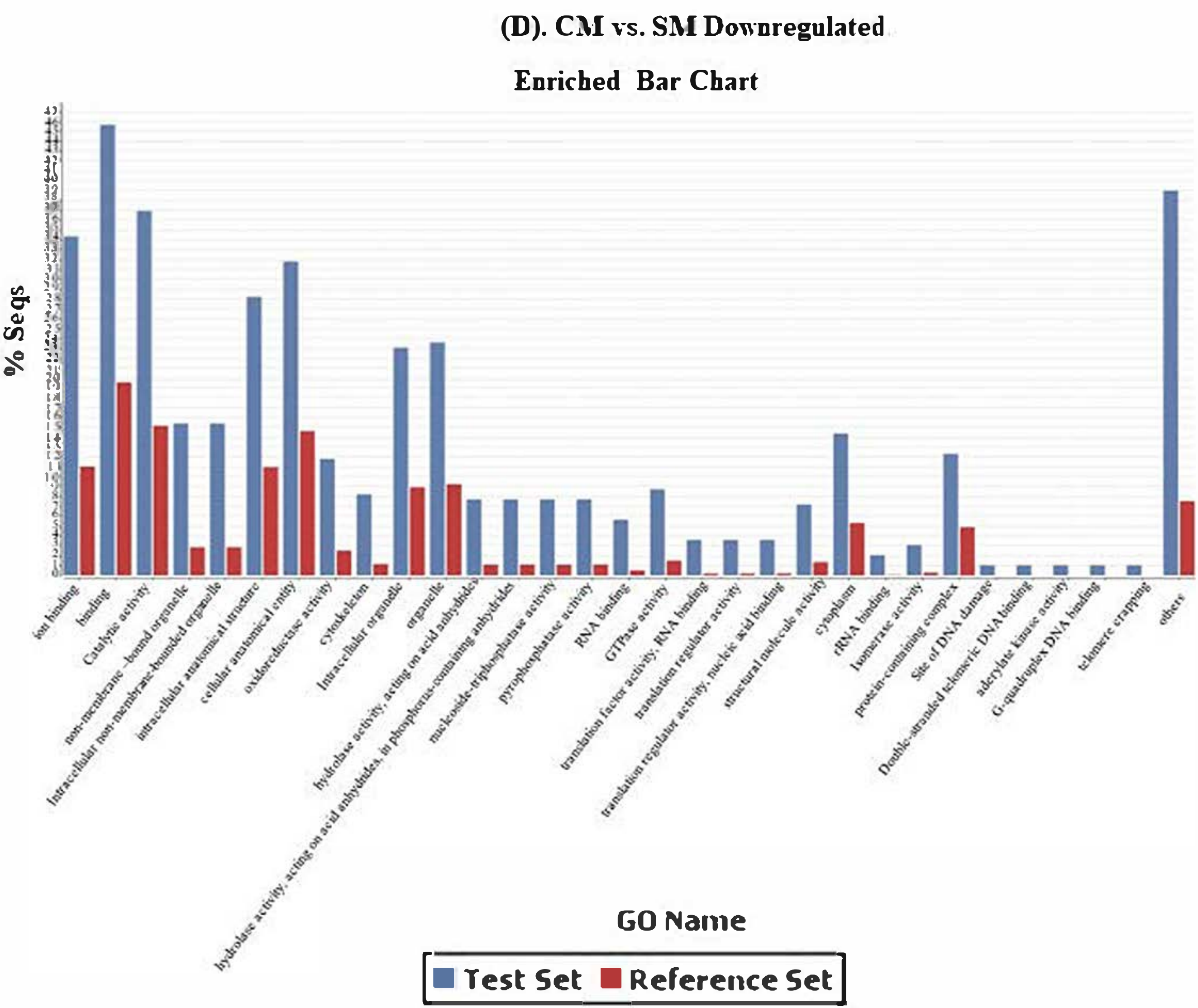

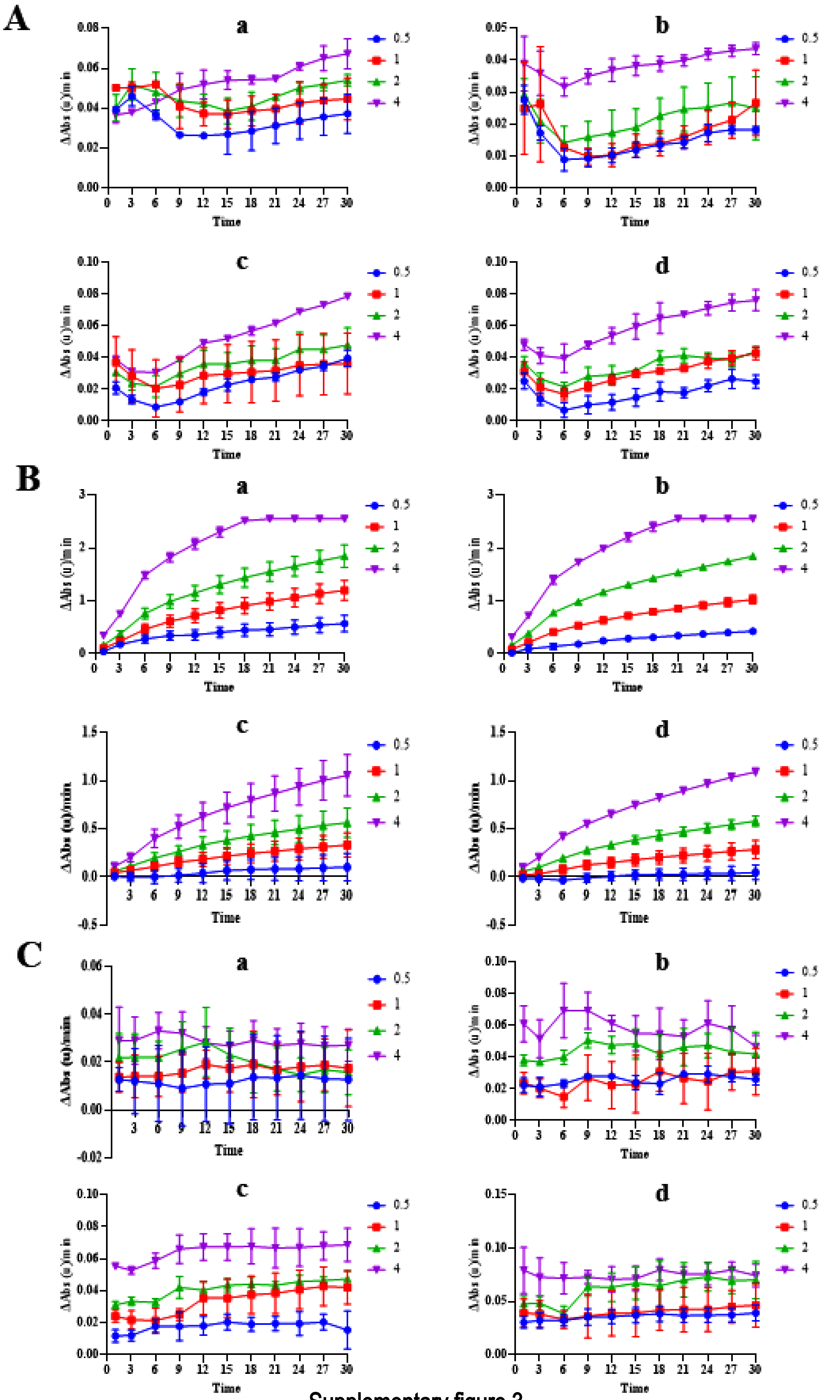

Supplementary figure 3
