# Supplementary material for: Comparative gut proteomics study revealing adaptive physiology of Eurasian spruce bark beetle, Ips typographus (Coleoptera: Scolytinae)
Source: Front Plant Sci. 2023 Nov 21;14:1157455. doi: 10.3389/fpls.2023.1157455 (PMC10703158; doi:10.3389/fpls.2023.1157455)
Supplement: Supplementary Table 1 — Genes and their primer sequences used for RT-qPCR. [file DataSheet_2.pdf]

Supplementary Table 1. Genes and their primer sequences used for qRT-PCR.

S. No.	Gene Name	Gene ID	Primer Name and Sequence	Primer Length (bp)	Tm (°C)	Amplicon length (bp)
1	Glutathione S-transferase (GST1)	Ityp05781	**GST1_F:** GTAGATCAGCGCCTCCATTT	20	60	136
			**GST1_R:** GGGACTGATAAGCTTGCACCACT	23		
2	Glutathione S-transferase-like (GST2)	Ityp14296	**GST2_F:** TGGAAAGCTCACTTGGGCCGA	21	60	133
			**GST2_R:** CCGTGGTATACTGACAACGTTCTCTCG	27		
3	Alcohol dehydrogenase [NADP (+)]-like (ADH6)	Ityp03643	**ADH6_F:** TCAACCAGGATCAAGTAAGAAGG	23	60	141
			**ADH6_R:** GATACGACACCACCACTATTCC	22		
4	Glucose dehydrogenase [acceptor] (GLD)	Ityp04644	**GLD_F:** GGCGACTGCAATGGAGTATT	20	60	106
			**GLD_R:** CAGGTTCGGGTCGTTGTATTT	21		
5	Fatty aldehyde dehydrogenase-like (FAldDH)	Ityp09529	**FALDH_F:** TGCTCCCGACTACGTTCTAT	22	60	109
			**FALDH_R:** AGTTGTCAGATTCCTTCGGATTT	18		
6	Hypothetical protein D910_04066 (SDH)	Ityp10381	**SDH_F:** GTTCCAGACATGACCAACTTCT	24	60	127
			**SDH_R:** CTCGATCATCCACGCTTTGT	22		
7	IDLDH_IPSPIRecName: Full= Ipsdienol dehydrogenase (IDLDH)	Ityp14703	**IDLDH_F:** GGAAGGCCAGAAAGGCATAA	23	60	115
			**IDLDH_R:** GGGATAGGTCAAGGAGAGGAT	25		
8	Serine protease inhibitor 27A-like (Serpin)	Ityp15930	**Serpin_F:** TTCAACGCTACTCACCCATTT	23	60	107
			**Serpin_R:** CCAATGTCCAGCTCCTGATAC	29		
9	Carboxypeptidase Q-like (CBQ)	Ityp14491	**CBQ_F:** CCTACAACGCCACCCATTAT	21	60	122
			**CBQ_R:** GATGATACAACCGCCCTTCTT	22		
10	Xaa-Pro aminopeptidase 1 (XPNPEP1)	Ityp16405	**XPNPEP1_F:** CTGGTGACGTGTTCCGTATT	22	60	134
			**XPNPEP1_R:** CTCGTTAGGGATGATGGAAGTG	21		
11	Lipase 3-like (Lipase)	Ityp00655	**Lipase_F:** GGCACTCAATGGGTACAACTAT	22	60	116
			**Lipase_R:** GCGCAAATGGGTCATGAAAG	22		
12	Esterase B1-like (Esterase 1)	Ityp08888	**Esterase1_F:** TTCCCTATGCAAAACCGCCCATTG	24	60	102
			**Esterase1_R:** CAGTCTTCAGAACCGCCTATT	21		
13	Esterase E4-like (Esterase 2)	Ityp10251	**Esterase2_F:** GTGGTGGTCAGTCAGAAGTTAG	22	60	109
			**Esterase2_R:** CAACTTTAGGCCACGTTATGTTATC	25		
14	Juvenile hormone esterase (JHE)	Ityp05867	**JHE_F:** CGACACATTGCGACGATTTAC	21	60	139
			**JHE_R:** GGATTTGGATTGCCCTGTTTG	21		

15	Acyl-coenzyme A thioesterase 13-like (ACOT13)	Ityp06393	**ACOT13_F:** GAAGTGCCTAGTGTTTCTGTA	21	60	165
			**ACOT13_R:** CCCTTTCACTAGAACTTCACCAGTAGC	27		
16	Venom carboxylesterase-6-like (Vcarboxy)	Ityp20267	**vCaE_F:** CAAATGGCTGAGCAACTATTATCC	21	60	133
			**vCaE_R:** CTGGACTGGAGTTGGGTTTAG	25		
17	Trypsin precursor (Trypsin)	Ityp09160	**Trypsin_F:** TGGCTGGTATCGTGTCTTTC	20	60	100
			**Trypsin_R:** GAGTTGTCCCTGATCCAGTTC	20		
18	Antitrypsin-like isoform X4 (Antitrypsin)	Ityp11942	**Antitrypsin_F:** ACCTAAGAAAGCAGCCTCAAA	21	60	149
			**Antitrypsin_R:** GGGTTTCCCAGATGCCTTTA	20		
19	NADPH: adrenodoxin oxidoreductase, mitochondrial (NADPH1)	Ityp00272	**NADPH1_F:** CGTATTCAGCCCGATGTTTCTA	27	60	102
			**NADPH1_R:** TCGTCTCCCTCCAACAAATTC	27		
20	NADH-ubiquinone oxidoreductase 75 kDa subunit, mitochondrial (NADPH2)	Ityp02701	**NADPH2_F:** GGTACAACTGGCAGAGGCAACG	22	60	137
			**NADPH2_R:** GCCTTGCAGTGAAACTGTACGGC	23		
21	40S ribosomal protein S3-A (**Internal control gene**) (RPS3)	Ityp04549	**RPS3-a_F:** GCCCCTTCAATGTTTGCCAC	20	60	119
			**RPS3-a_R:** AAGTCCGCCAAACTAACCTCA	21		
